# PAC–Bayes Guarantees for Data-Adaptive Pairwise Learning

**DOI:** 10.3390/e27080845

**Published:** 2025-08-08

**Authors:** Sijia Zhou, Yunwen Lei, Ata Kabán

**Affiliations:** 1 School of Computer Science, University of Birmingham, Edgbaston, Birmingham, B15 2TT, UK; 2 Department of Mathematics, University of Hong Kong, Pokfulam, Hong Hong, China

**Keywords:** pairwise learning, randomized algorithms, PAC–Bayes, algorithmic stability

## Abstract

We study the generalization properties of stochastic optimization methods under adaptive data sampling schemes, focusing on the setting of pairwise learning, which is central to tasks like ranking, metric learning, and AUC maximization. Unlike pointwise learning, pairwise methods must address statistical dependencies between input pairs—a challenge that existing analyses do not adequately handle when sampling is adaptive. In this work, we extend a general framework that integrates two algorithm-dependent approaches—algorithmic stability and PAC–Bayes analysis for this purpose. Specifically, we examine (1) Pairwise Stochastic Gradient Descent (Pairwise SGD), widely used across machine learning applications, and (2) Pairwise Stochastic Gradient Descent Ascent (Pairwise SGDA), common in adversarial training. Our analysis avoids artificial randomization and leverages the inherent stochasticity of gradient updates instead. Our results yield generalization guarantees of order $n^{-1/2}$ under non-uniform adaptive sampling strategies, covering both smooth and non-smooth convex settings. We believe these findings address a significant gap in the theory of pairwise learning with adaptive sampling.

## 1. Introduction

The increasing availability of data makes it feasible to use increasingly large models in principle. However, this comes at the expense of an increasing computational cost of training these models in large pairwise learning applications. Some notable examples of pairwise learning problems include ranking and preference prediction, AUC maximization, and metric learning [1,2,3,4,5]. For instance, in metric learning we aim to learn an appropriate distance or similarity to compare pairs of examples, which has numerous applications such as face verification, person re-identification (Re-ID) [6,7,8,9,10], and bioactivity prediction [11]. Pairwise learning has also been applied to positive-unlabeled (PU) learning problems [12], where only positive and unlabeled examples are available. Such problems arise in one-class classification settings, with practical applications in areas such as fault detection and diagnosis in advanced engineering systems [13]. Given the broad relevance of pairwise learning, there is a pressing need to deepen our theoretical understanding of its generalization properties. This in turn can inform the design of algorithms that generalize reliably to unseen pairs and offer interpretability and trustworthiness to end users.

In both pointwise and pairwise learning settings, Stochastic Gradient Descent (SGD) and Stochastic Gradient Descent Ascent (SGDA) are widely used for large-scale minimization and min-max optimization problems in machine learning due to their favorable computational efficiency. These methods rely on stochastic sampling strategies to approximate the true gradients, and several works have explored data-dependent sampling techniques to accelerate convergence to the optimum [14,15,16,17,18,19]. SGDA, in particular, is a standard approach in solving min-max problems, finding notable applications in generative adversarial networks (GANs) [20] and adversarial training [21,22].

Adversarial perturbations are subtle, often imperceptible modifications to input data designed to deceive models and cause incorrect predictions [23]. Recent studies in pairwise learning have explored strategies to enhance adversarial robustness, applying adversarial pairwise learning methods to min-max problems across various domains, such as metric learning [24,25], ranking [25,26], and kinship verification [27]. These developments illustrate the need for robust, theoretically grounded pairwise methods that can withstand adversarial attacks while maintaining generalization performance.

Under the assumption of i.i.d. data points in classic pointwise learning, the empirical risk of a fixed hypothesis is an average of i.i.d. random variables. However, in pairwise learning the pairs derived from i.i.d. points are no longer i.i.d. Instead, when the loss is symmetric and computed over all unordered pairs, the empirical risk takes the form of a second-order *U*-statistic. Therefore, results on *U*-processes may be used to investigate the generalization analysis of pairwise learning [3,28]. While there is much research on the generalization analysis of pairwise learning, the effect of non-uniform, data-dependent sampling schemes has not been rigorously studied.

Non-uniform sampling can be beneficial in noisy data situations where the training examples may not be equally reliable or equally informative. Some examples may be less important than others, or even misleading—e.g., mislabeled examples or examples situated in an ambiguous class-overlap region. In rare cases when the usefulness or importance of individual training examples is known, then the sampling distribution can be designed and fixed before training, and this may improve the representativeness of the sample. For instance, in the case of infrequent observations [29], inverse frequency sampling prioritizes rare examples that may be underrepresented in the training set, ensuring their proper influence. However, in most cases the relative importance of training examples is not known a priori; hence, it is desirable to learn the sampling distribution together with training the model.

The idea of adaptive sampling refers to a sampling distribution that depends on the training sample. Such non-uniform and data-dependent sampling shows great potential in the literature of randomized algorithms for both SGD- [14] and SGDA-based [30] optimizers, in both pointwise and pairwise settings. Importance sampling [14] is one of the widely used of such strategies, and a few others will be reviewed shortly in Section 2. Therefore, recent work [31,32] has begun to develop a better understanding of the generalization behavior of such algorithms, which we continue here in the setting of pairwise learning. The main bottlenecks in the analysis of adaptive sampling-based stochastic optimizers are that (i) a correction factor is often used to ensure the unbiasedness of the gradient [14], which also depends on training data points complicating the analysis, and (ii) in the pairwise setting we also need to cater to statistical dependencies between data pairs, which are due to the fact that each point participates in multiple pairs.

To tackle these problems, we develop a PAC–Bayesian analysis of the generalization of pairwise stochastic optimization methods, which removes the need for a correction factor, and we use *U*-statistics to capture the statistical structure of pairwise loss functions. The PAC–Bayes framework allows us to obtain generalization bounds that hold uniformly for all posterior sampling schemes, under a mild condition required on a pre-specified prior sampling scheme (chosen as the uniform sampling). For randomized methods, such as Pairwise SGD and Pairwise SGDA, the sampling index pairs will be treated as hyperparameters that follow a sampling distribution.

Our main contributions are listed in Table 1, summarizing the generalization bounds of the order $\tilde{O}\left(1/\sqrt{n}\right)$ for these randomized algorithms under different assumptions, where *n* is the sample size.

Our technical contributions are summarized as follows:

- We bound the generalization gap of randomized pairwise learning algorithms that operate with an arbitrary data-dependent sampling, in a PAC–Bayesian framework, under a sub-exponential stability condition.
- We apply the above general result to Pairwise SGD and Pairwise SGDA with arbitrary sampling. For both of these algorithms, we verify the sub-exponential stability in both smooth and non-smooth problems.
- We exemplify how our bounds can inform algorithm design, and we demonstrate how to extract meaningful information from the resulting algorithms.

Our work builds on well-established tools, including a specific flavor of PAC–Bayesian analysis [31], U-statistic decomposition, and a moment bound for uniformly stable pairwise learning algorithms [33], aimed at bringing theoretical insight into an important, yet relatively underexplored, setting: pairwise learning with adaptive sampling. To the best of our knowledge, our analysis is the first to derive explicit generalization bounds for this setting.

The remainder of the paper is organized as follows. We survey the related work on the generalization analysis and non-uniform sampling in Section 2. We give a brief background on *U*-statistics and algorithmic stability analysis in Section 3. Our general result and its applications to Pairwise SGD and Pairwise SGDA are presented in Section 4.

## 2. Related Work 

**Adaptive Sampling in Stochastic Optimization**. Importance Sampling for Stochastic Gradient Descent was proposed in [14]. To compute the stochastic gradient, a training example $z_{i}$ (i∈[n]) is sampled with probability proportional to the gradient norm $p_{i}\propto ∥\nabla_{w}\ell \left(w;z_{i}\right)∥$, where w are the model parameters and *ℓ* is the loss function. This prioritizes high-impact updates from the perspective of optimization—the authors proved that this can significantly reduce the variance of the stochastic gradient and accelerate convergence to the optimum. A related idea, loss-based sampling, proposed in [16], assigns sampling probabilities proportional to the loss evaluated on training points, that is, $p_{i}\propto \ell \left(w;z_{i}\right)$, thereby focusing on hard-to-fit examples. The authors show faster convergence to the optimum. While these works do not consider pairwise learning, they represent landmarks on adaptive sampling in stochastic optimization.

Furthermore, there are variants of these ideas aimed at lightening the computational demand. These include upper bounds to the gradient norm, shown to exhibit better performance in comparison with the loss-based sampling [34]. The work in [18] proposes to sample the training points based on their relative distance to each other. Another more recent data-dependent sampling approach called group sampling appears in [19] and has been applied to a person re-identification (Re-ID) application.

Adaptive sampling is an umbrella term referring to sampling distributions that depend on the training sample. Here, we mentioned a few of the most prominent existing examples. However, the appropriate sampling distribution is task-dependent. The works discussed, and most of the previous work on adaptive sampling in stochastic optimization, aim at accelerating convergence to the optimum. Therefore, they have no explicit cost for data-dependent sampling; instead, they have a multiplicative correction factor to ensure an unbiased gradient. However, the goal of learning is generalization, which is different from achieving the global optimum on training data. There must be a cost for data-dependent sampling to avoid over-reliance on a biased subset of points. Our forthcoming generalization bounds will quantify this rigorously and provide guidance for algorithm design.

With the exception of [32] and our previous conference paper [31], results on the generalization analysis of the resulting randomized algorithms are very scarce, which is our goal to advance in this paper specifically for the pairwise learning setting.

**Generalization through Algorithmic Stability**. Stability was popularized in the seminal work of [35], to formalize the intuition that algorithms whose output is resilient to changing an example in its input data will generalize. The stability framework subsequently motivated a chain of analysis of randomized iterative algorithms, such as SGD [36] and SGDA [37,38]. While the stability framework in the previous work is well suited for SGD-type algorithms that operate a uniform sampling scheme [36], this framework alone is unable to tackle arbitrary data-dependent sampling schemes.

**Generalization through PAC–Bayes**. The PAC–Bayes theory of generalization is another algorithm-dependent framework in statistical learning, the gist of which is to leverage a pre-specified prior distribution on the parameters of interest to obtain generalization bounds that hold uniformly for all posterior distributions [39,40]. Its complementarity with the algorithmic stability framework sparked ideas for combining them [41,42,43,44], some of which are also applicable to randomized learning algorithms such as SGD and SGLD [32,45,46,47]. While insightful, these works assume i.i.d. examples and cannot be applied to non-i.i.d. settings that arise in pairwise learning.

In non-i.i.d. settings, ref. [48] gave PAC–Bayes bounds using fractional covers, which allows for handling the dependencies within the inputs. This gives rise to generalization bounds for pairwise learning, with predictors following a distribution induced by a prior distribution on the model’s parameters. However, with SGD-type methods in mind, which have a randomization already built into the algorithm, the classic PAC–Bayes approach of placing a prior on a model’s parameters would be somewhat artificial. Indeed, considerable research effort has been spent to reverse such randomization [49]. Another issue concerns the prior specification—recent research [50] reveals that placing sufficient prior mass on good predictors is a condition for meaningful PAC–Bayes guarantees. These are difficult to set without a strong prior knowledge. Instead, the construction proposed in [31,32] (albeit restricted to the i.i.d. setting) is to exploit this built-in stochasticity of modern gradient-based optimization algorithms directly, by interpreting it as a PAC–Bayes prior placed on a hyperparameter. We will build on this idea further in this work.

## 3. Preliminaries

### 3.1. Pairwise Learning and U-Statistics

Let D be an unknown distribution on sample space Z. We denote by $W\subseteq R^{d}$ the parameter space, and Φ will be a hyperparameter space. Given a training set $S=\{z_{1},\ldots ,z_{n}\}$ drawn i.i.d. from D and a hyperparameter ϕ∈Φ, a learning algorithm *A* returns a model parameterized by A(S;ϕ)∈W.

We are interested in pairwise learning problems and will use a pairwise loss function $\ell :W\times \left(Z\times Z\right)\mapsto R_{+}$ to measure the mismatch between the prediction of the model that acts on example pairs. The generalization error, or true risk, is defined as the expected loss of the learned predictor applied on an unseen pair of inputs drawn from $D^{2}$, that is,

(1) $$R\left(A\left(S;\phi \right)\right):=E_{z,\tilde{z}∼D}\left[\ell \left(A\left(S;\phi \right),z,\tilde{z}\right)\right].$$

Since D is unknown, we consider the empirical risk,

(2) $$R_{S}\left(A\left(S;\phi \right)\right):=\frac{1}{n(n-1)}\sum_{i,j\in [n]:i\neq j}\ell \left(A\left(S;\phi \right),z_{i},z_{j}\right),$$

where [n]:={1,…,n}. The generalization error is a random quantity as a function of the sample *S*, which does not consider the randomization used when selecting the data or feature index for the update rule of *A* at each iteration.

To take advantage of the built-in stochasticity of the type of algorithms we consider, we further define two distributions on the hyperparameter space Φ: a sample-independent distribution P and a sample-dependent distribution Q. In this stochastic or randomized learning algorithm setting, the expected risk and the expected empirical risk (both with respect to Q) are defined as

$$R\left(Q\right)=\underset{\phi ∼Q}{E}\left[R\left(A\left(S;\phi \right)\right)\right],\;R_{S}\left(Q\right)=\underset{\phi ∼Q}{E}\left[R_{S}\left(A\left(S;\phi \right)\right)\right].$$

We denote the difference between the risk and the empirical risk (i.e., the generalization gap) by $G\left(S,\phi \right):=R\left(A\left(S;\phi \right)\right)-R_{S}\left(A\left(S;\phi \right)\right)$.

The difficulty with the pairwise empirical loss (2) is that, even with *S* consisting of i.i.d. instances, the pairs from *S* are dependent of each other. Instead, $R_{S}\left(A\left(S;\phi \right)\right)$ is a second-order *U*-statistic. A powerful technique to handle the *U*-statistic is the representation as an average of “sums-of-i.i.d.” blocks [28]. That is, for a symmetric kernel q:Z×Z↦R, we can represent the *U*-statistic $U_{n}:=\frac{1}{n(n-1)}\sum_{i,j\in [n]:i\neq j}q\left(z_{i},z_{j}\right)$ as

(3) $$\begin{array}{c} U_{n}=\frac{1}{n!}\sum_{\sigma }\frac{1}{\left\lfloor n/2\right\rfloor }\sum_{i=1}^{\left\lfloor n/2\right\rfloor }q\left(z_{\sigma (i)},z_{\sigma (\left\lfloor \frac{n}{2}\right\rfloor +i)}\right), \end{array}$$

where σ ranges over all permutations of {1,…,n}.

### 3.2. Connection with the PAC–Bayesian Framework

As described above, we consider two probability distributions on the hyperparameter space Φ, to account for the stochasticity in stochastic optimization algorithms, such as Pairwise SGD and Pairwise SGDA, where the hyperparameter ϕ∈Φ is a sequence of pairs of indices that follow a discrete distribution. For instance, in Pairwise SGD, in every iteration t∈[T], we have $\phi_{t}=\left(i_{t},j_{t}\right)$, that is, a pair of independently sampled sample indices, drawn from $\left\{\left(i_{t},j_{t}\right):i_{t},j_{t}\in \left[n\right],i_{t}\neq j_{t}\right\}$ with replacement (more details in Section 4.1). We define two distributions over Φ, namely the PAC–Bayes prior P, which needs to be specified before seeing the training data, and the PAC–Bayes posterior Q, which is allowed to depend on the training sample. This setting is different from the classic use of PAC–Bayes, which defines the two distributions directly on the trainable parameter space W. Our distributions defined on Φ indirectly induce distributions on the parameter estimates, without the need to know their parametric form. This setting of PAC–Bayes was formerly introduced in London [32] in combination with algorithmic stability and further improved in our previous work [31], both restricted to the i.i.d. pointwise setting.

### 3.3. Connection with the Algorithmic Stability Framework

A more recent framework for the generalization problem considers algorithmic stability [35], which measures the sensitivity of a learning algorithm to small changes in the training data. The concept considered in our work among several notions of algorithmic stability is uniform stability.

**Definition** **1** (Uniform Stability). *For ∀ϕ, we say an algorithm A:S↦A(S;ϕ) is $\beta_{\phi }$-uniformly stable if*

(4) $$|\ell \left(A\left(S;\phi \right),z,\tilde{z}\right)-\ell \left(A\left(S^{\prime },\phi \right),z,\tilde{z}\right)|\leq \beta_{\phi },\;\forall z,\tilde{z}\in Z,$$

*where $S,S^{\prime }\in Z^{n}$ differs by at most a single example.*

The algorithmic stability framework is suitable for analyzing certain deterministic learning algorithms, or randomized algorithms with a pre-defined randomization. In turn, here we are concerned with inherently stochastic algorithms where we wish to allow any data-dependent stochasticity, such as the variants of importance sampling and other recent practical methods mentioned in the related works, e.g., [14,15,18,19,34]. Moreover, in principle our framework and results are applicable even if the sampling distribution is learned from the training data itself.

**Sub-exponential Stability**. A useful definition of stability that captures the stochastic nature of the algorithms we are interested in is the sub-exponential stability introduced in Zhou et al. [31]. Recall that ϕ is a random variable following a distribution defined on Φ. Therefore, the stability parameter $\beta_{\phi }$ is also a random variable as a function of ϕ. We want to control the tail behavior of $\beta_{\phi }$ around a value that decays with the sample size *n*, and we define the sub-exponential stability as the following.

**Definition** **2** (Sub-exponential stability). *Fix any prior distribution P on $\Phi =\prod_{t=1}^{T}\Phi_{t}$. We say that a stochastic algorithm is sub-exponentially $\beta_{\phi }$-stable (with respect to P) if, given any fixed instance of ϕ∼P, it is $\beta_{\phi }$-uniformly stable and there exist $c_{1},c_{2}\in R$ such that for any δ∈(0,1/n], the following holds with probability of at least 1−δ:*

(5) $$\beta_{\phi }\leq c_{1}+c_{2}\log\left(1/\delta \right).$$

## 4. Main Results

In this section, we will give generalization bounds for Pairwise SGD and Pairwise SGDA in pairwise learning. To this aim, we first give a general result (Lemma 1) to show the connection between the sub-exponential stability condition (Assumption 2) and the generalization gap in the case of pairwise learning. We then derive stability bounds to show that this assumption holds for Pairwise SGD and Pairwise SGDA, in both smooth convex and non-smooth convex cases. Based on these, we apply the stability bounds to Lemma 1 to derive the corresponding generalization bounds. We use $K≲K^{\prime }$ if there exists a universal constant a>0 such that $K\leq aK^{\prime }$. The proof is given in Appendix A.

**Lemma** **1** (Generalization of randomized pairwise learning). *Given distribution P, $c_{1},c_{2}>0$, and M-bounded loss for a sub-exponentially stable algorithm A, ∀δ∈(0,1/n), with probability of at least 1−δ, the following holds uniformly for all Q absolutely continuous with respect to P:*

$$E_{\phi ∼Q}\left[G(S,\phi )\right]≲\left(\mathrm{KL}\left(Q∥P\right)+\log\frac{1}{\delta }\right)\max\left\{c_{1}\log n+c_{2}\log^{2}n,\frac{M}{\sqrt{n}}\right\},$$

*where KL(Q∥P) is the KL divergence between Q and P, $\mathrm{KL}\left(Q∥P\right)=\int_{\phi \in \Phi }\log\frac{dQ}{dP}dQ$.*

A strength of Lemma 1 is that we only need to check the sub-exponential stability condition under a prior distribution P, and  Lemma 1 automatically translates it to generalization bounds for any posterior distribution Q.

In the forthcoming applications both Q and *P* are discrete distributions, so we have $\mathrm{KL}\left(Q∥P\right)=\sum_{\phi \in \Phi }Q\left(\phi \right)\log\frac{Q(\phi )}{P(\phi )}$. In particular, the prior P will be most naturally chosen as the discrete uniform distribution in the context of applications to stochastic optimization in Section 4.2. Let P=U with U denoting the uniform distribution on ${\left(\left[n\right]\times \left[n\right]\right)}^{T}$. Hence, the absolute continuity condition is satisfied, ensuring that KL(Q∥P)<∞ for all distributions Q over the set ${\left(\left[n\right]\times \left[n\right]\right)}^{T}$. Furthermore, in this setting, we have KL(Q∥P)=−H(Q)+2Tlogn, where H denotes the Shannon entropy.

We introduce some classic assumptions that are frequently employed in the analysis of randomized algorithms. Let ${∥\cdot ∥}_{2}$ denote the Euclidean norm. Let *S* and $S^{\prime }$ be neighboring datasets (i.e., they differ in only one example, which we denote as the *k*-th example, k∈[n]). For brevity, we write ℓ(w) for $\ell (w;z,\tilde{z})$, where we mean a property that holds for all $z,\tilde{z}\in Z$.

**Assumption** **1.** *Let L>0. We say ℓ is L-Lipschitz if for any $w_{1}$, $w_{2}$∈W, we have $|\ell \left(w_{1}\right)-\ell \left(w_{2}\right)|\leq L∥w_{1}-w_{2}{∥}_{2}.$*

**Assumption** **2** (Convexity).
*We say ℓ is convex if the following holds $\forall w_{1},w_{2}\in W$:*

$$\ell \left(w_{1}\right)\geq \ell \left(w_{2}\right)+\langle \nabla \ell \left(w_{2}\right),w_{1}-w_{2}\rangle ,$$

*where 〈·,·〉 represents the inner product.*

**Assumption** **3.** *Let α≥0. We say a differentiable function ℓ is α-smooth, if for any $w_{1}$, $w_{2}$∈W, $∥\nabla \ell \left(w_{1}\right)-\nabla \ell \left(w_{2}\right){∥}_{2}\leq \alpha {∥w_{1}-w_{2}∥}_{2},$ where ∇ℓ represents the gradient of ℓ.*

### 4.1. Stability and Generalization of Pairwise SGD

We now consider Pairwise SGD, which, as we will show, also satisfies the sub-exponential stability condition in both smooth and non-smooth cases.

We denote $w_{1}$ an initial point and a uniform distribution over ${\left([n]\times [n]\right)}^{T}$. At the *t*-th iteration for Pairwise SGD, a pair of sample indices $\phi_{t}=\left(i_{t},j_{t}\right)$ is uniformly randomly selected from the set $\left\{\left(i_{t},j_{t}\right):i_{t},j_{t}\in \left[n\right],i_{t}\neq j_{t}\right\}$. This forms a sequence of index pairs $\phi =(\phi_{1},\ldots ,\phi_{T})$. For step size $\eta_{t}$, the model is updated by $w_{t+1}=w_{t}-\eta_{t}\nabla \ell \left(w_{t};z_{i_{t}},z_{j_{t}}\right).$

The following lemma shows that Pairwise SGD with uniform sampling applied to both smooth and non-smooth problems enjoys sub-exponential stability. The proof is given in Appendix B.1.

**Lemma** **2** (Sub-exponential stability of Pairwise SGD).
*Let $\left\{w_{t}\right\},\left\{w_{t}^{\prime }\right\}$ be two parameter sequences produced by Pairwise SGD with fixed step sizes and uniform sampling P, while being trained on neighboring training samples S and $S^{\prime }$. Suppose there is a loss in Lipschitzness and convexity (i.e., Assumptions 1 and 2 hold). Then, we have the following:*

*(1)*  *At the t-th iteration, we have sub-exponential stability (Definition 2) with*
$$c_{1}\;=\;2\sqrt{e}L^{2}\eta \left(\sqrt{t}+2t/n\right)\;\mathrm{and}\;c_{2}\;=\;4\sqrt{e}L^{2}\eta \left(1+2{\left(t/n\right)}^{\frac{1}{2}}\right).$$
*(2)*  *If in addition the loss is also smooth (Assumption 3 holds), then with step size η≤2/α, at the t-th iteration, we have sub-exponential stability (Definition 2) with*
$$c_{1}=4L^{2}\eta t/n\;\mathrm{and}\;c_{2}=4L^{2}\eta \left(1+2{\left(t/n\right)}^{\frac{1}{2}}\right).$$

Using Lemmas 1 and 2, we obtain the following generalization bound for Pairwise SGD with general sampling.

**Theorem** **1** (Generalization bounds for Pairwise SGD).
*Assume ℓ is M-bounded, Lipschitz, and convex (cf. Assumptions 1 and 2). For any δ∈(0,1), Pairwise SGD with fixed step sizes satisfies the following generalization guarantees with probability of at least 1−δ over S, $S∼D^{n}$, for all posterior sampling distributions Q on ${\left([n]\times [n]\right)}^{T}$:*

*(1)*  *After T iterations, we have*
$$E_{Q}\left[G(S,\phi )\right]\;≲\;\left(\;T\log n\;-\;H\left(Q\right)\;+\;\log\frac{1}{\delta }\;\right)\;\max\;\left\{\;L\eta \left(\sqrt{T}\;+\;\frac{T}{n}\;+\;\sqrt{\frac{T}{n}}\right)\log^{2}n,\frac{M}{\sqrt{n}}\;\right\}.$$
*(2)*  *If in addition the loss is also smooth (Assumption 3 holds), then with step size η≤2/α, we have*
$$E_{Q}\left[G(S,\phi )\right]≲\left(\;T\log n\;-\;H\left(Q\right)\;+\;\log\frac{1}{\delta }\;\right)\max\left\{L\eta \left(\frac{T}{n}\;+\;1\;+\;\sqrt{\frac{T}{n}}\right)\log^{2}n,\frac{M}{\sqrt{n}}\right\}.$$

**Remark** **1.** *Suppose $\mathrm{KL}(Q∥U)\in \tilde{O}\left(1\right)$, as it has been tacitly assumed also in previous work [32] when quantifying the generalization convergence rate. Taking the choice of parameters suggested by [51], if $\eta =\Theta (T^{-\frac{3}{4}})$ and $T=\Theta (n^{2})$ in the non-smooth case (part 1), then the above theorem implies bounds of the order $\tilde{O}\left(1/\sqrt{n}\right)$. In the smooth case (part 2), an analysis of the trade-off between optimization and generalization, Lei et al. [33] suggested setting T=Θ(n) and $\eta =\Theta (1/\sqrt{T})$ to get a Pairwise SGD to iterate with a good generalization performance. With these choices, our bounds in Theorem 1 are of order $\tilde{O}\left(1/\sqrt{n}\right)$, which are not improvable in general.*

**Remark** **2** (Implication of the $\mathrm{KL}\left(Q∥U\right)=\tilde{O}\left(1\right)$ assumption). *Let supp(Q)⊆Φ denote the support of Q, where $\Phi ={\left(\left[n\right]\times \left[n\right]\right)}^{T}$ in pairwise learning.*

*Since $U\left(\phi \right)=1/n^{2T}$ for all ϕ, the KL divergence is*

$$\mathrm{KL}\left(Q∥U\right)=\sum_{\phi \in \Phi }Q\left(\phi \right)\log\left(\frac{Q(\phi )}{1/n^{2T}}\right)=-H\left(Q\right)+2T\log n.$$

*To ensure this is of order $\tilde{O}\left(1\right)$, we need $-H\left(Q\right)+2T\log n\leq \tilde{O}\left(1\right)$; hence,*

(6) $$\begin{array}{c} H\left(Q\right)\geq 2T\log n-\tilde{O}\left(1\right)=H\left(U\right)-\tilde{O}\left(1\right). \end{array}$$

*To give more intuition, consider Q on a restricted support. This is very much a worst-case scenario, as it would imply completely discarding (rather than down-weighting) some of the training points. For such Q, the maximum entropy occurs when Q is uniform over its support, so $Q\left(\phi \right)=\frac{1}{\left|\mathrm{supp}(Q)\right|}$, and $\mathrm{KL}\left(Q∥U\right)=\log\left(\frac{n^{2T}}{\left|\mathrm{supp}(Q)\right|}\right)$. In this case, having $\mathrm{KL}\left(Q∥U\right)=\tilde{O}\left(1\right)$ requires*

(7) $$\begin{array}{c} \left|\mathrm{supp}\left(Q\right)\right|=\Omega \left(\frac{n^{2T}}{\mathrm{poly}(T,n)}\right). \end{array}$$

*To sum up, Q must satisfy the entropy lower bound (6), and to achieve that entropy on a restricted support, it must have a large enough support, i.e., at least a Ω(1/poly(T,n)) fraction of the entire *Φ.

*In Pairwise SGD with non-uniform data-dependent sampling, this result tells us that in order to keep generalization rates that compare against the uniform baseline, Q cannot discard a large subset of the index sequences. This limits how aggressively one can compress or “distill” a dataset (as in core-set selection or dataset distillation) without paying a KL penalty that slows down the rate—at least as long as the prior is uniform.*

Non-uniform sampling alone is insufficient for robust learning. While it may mitigate the effect of a small fraction of bad examples (e.g., out-of-distribution or mislabeled training examples), achieving robustness also requires modeling choices such as robust loss functions. In the next section we approach this via Pairwise SGDA, the type of optimization required in adversarially robust training.

### 4.2. Stability and Generalization of Pairwise SGDA

In this subsection, we discuss Pairwise SGDA for solving minimax problems in the convex-concave case. We will abuse the notations to apply them to the minimax case. We receive a model $A\left(S;\phi \right):=\left(A_{w}\left(S;\phi \right),A_{v}\left(S;\phi \right)\right)\in W\times V$ by applying a learning algorithm *A* on training set *S* and measure the performance with respect to a loss function $\ell :\left(w,v\right)\mapsto \ell \left(w,v;z,\tilde{z}\right)$. For any ϕ∈Φ, we consider the risk defined as

$$\min_{w\in W}\max_{v\in V}R\left(A_{w}\left(S;\phi \right),A_{v}\left(S;\phi \right)\right):=E_{z,\tilde{z}∼D}\left[\ell \left(A_{w}\left(S;\phi \right),A_{v}\left(S;\phi \right);z,\tilde{z}\right)\right].$$

We consider the following empirical risk as the approximation:

$$R_{S}\left(A_{w}\left(S;\phi \right),A_{v}\left(S;\phi \right)\right):=\frac{1}{n(n-1)}\sum_{i,j\in [n]:i\neq j}\ell \left(A_{w}\left(S;\phi \right),A_{v}\left(S;\phi \right);z_{i},z_{j}\right).$$

We consider Pairwise SGDA with a general sampling scheme, where the random index pairs follow from a general distribution.

We denote $w_{1}$ and $v_{1}$ the initial points. Let $\nabla_{w}\ell$ and $\nabla_{v}\ell$ be the gradients with respect to w and v, respectively. Let P be a uniform distribution over ${\left([n]\times [n]\right)}^{T}$ and *S* be a training dataset with *n* samples. Let $\left(i_{t},j_{t}\right)$ from set $\left\{\left(i_{t},j_{t}\right):i_{t},j_{t}\in \left[n\right],i_{t}\neq j_{t}\right\}$ be drawn uniformly at random. At the *t*-th iteration, with step size sequence $\left\{\eta_{t}\right\}$, Pairwise SGDA updates the model as follows:

$$\left\{\begin{array}{c} w_{t+1}=w_{t}-\eta_{t}\nabla_{w}\ell \left(w_{t},v_{t};z_{i_{t}},z_{j_{t}}\right),\\ v_{t+1}=v_{t}+\eta_{t}\nabla_{v}\ell \left(w_{t},v_{t};z_{i_{t}},z_{j_{t}}\right). \end{array}\right.$$

Before giving the results for Pairwise SGDA, we restate the assumptions we need, adapted to the new setting with two distinct parameter vectors w and v [38,52].

**Assumption** **4.** *Let L≥0. We say a differentiable function ℓ is L-Lipschitz with respect to w and v if the following holds: For any $z,\tilde{z}\in Z$, w∈W, v∈V, we have*

$${∥\nabla_{w}\ell \left(w,v;z,\tilde{z}\right)∥}_{2}\leq L\;\mathrm{and}\;{∥\nabla_{v}\ell \left(w,v;z,\tilde{z}\right)∥}_{2}\leq L.$$

**Assumption** **5.** *Let α>0. We say a differentiable function ℓ is α-smooth if the following inequality holds for any $w_{1}$, $w_{2}\in W$, $v_{1}$, $v_{2}\in V$ and $z,\tilde{z}\in Z$:*

$${∥\left(\begin{array}{c} \nabla_{w}f\left(w_{1},v_{1};z,\tilde{z}\right)-\nabla_{w}f\left(w_{2},v_{2};z,\tilde{z}\right)\\ \nabla_{v}f\left(w_{1},v_{1};z,\tilde{z}\right)-\nabla_{v}f\left(w_{2},v_{2};z,\tilde{z}\right) \end{array}\right)∥}_{2}\leq \alpha {∥\left(\begin{array}{c} w_{1}-w_{2}\\ v_{1}-v_{2} \end{array}\right)∥}_{2}.$$

**Assumption** **6** (Convexity-Concavity).
*We say ℓ is concave if −ℓ is convex. We say ℓ is convex-concave if ℓ(·,v) is convex for every v∈V and ℓ(w,·) is concave for every w∈W.*

Now, we apply Lemma 1 to develop bounds for Pairwise SGDA in both smooth and non-smooth cases. In the following lemma, proved in Appendix B.2, we establish the sub-exponential stability of Pairwise SGDA.

**Lemma** **3** (Sub-exponential stability of Pairwise SGDA).
*Let $\left\{w_{t},v_{t}\right\},\left\{w_{t}^{\prime },v_{t}^{\prime }\right\}$ be two parameter sequences produced by Pairwise SGDA with fixed step sizes and uniform sampling P while being trained on neighboring samples S and $S^{\prime }$. Suppose the loss is Lipschitz and convex (cf. Assumptions 4–6). Then, we have the following:*

*(1)*  *At the t-th iteration, we have sub-exponential stability (Definition 2) with*
$$c_{1}=2\sqrt{2e}L^{2}\eta \left(\sqrt{t}+2t/n\right)\;\mathrm{and}\;c_{2}=4\sqrt{2e}L^{2}\eta \left(1+\sqrt{2t/n}\right).$$
*(2)*  *If in addition the loss is also smooth (cf. Assumption 5), then at the t-th iteration, sub-exponential stability (Definition 2) holds with*
$$c_{1}=4\sqrt{e}L^{2}\eta \exp\left(\frac{1}{2}\alpha^{2}t\eta^{2}\right)\left(1+2t/n\right)\;\mathrm{and}\;c_{2}=8\sqrt{e}L^{2}\eta \exp\left(\frac{1}{2}\alpha^{2}t\eta^{2}\right)\left(1+\sqrt{2t/n}\right).$$

We combine the above lemma with Lemma 1 to obtain bounds for Pairwise SGDA with a general sampling distribution.

**Theorem** **2** (Generalization bounds for Pairwise SGDA).
*Assume ℓ is M-bounded, Lipschitz, and convex (cf. Assumptions 4 and 6). For  any δ∈(0,1), Pairwise SGDA with fixed step sizes satisfies the following generalization guarantees with probability of at least 1−δ over draws of $S∼D^{n}$, for all posterior sampling distributions Q on ${\left([n]\times [n]\right)}^{T}$:*

*(1)*  *After T iterations, we have*
$$E_{\phi ∼Q}\left[G(S,\phi )\right]≲\left(\;T\log n\;-\;H\left(Q\right)\;+\;\log\frac{1}{\delta }\;\right)\max\left\{L^{2}\eta \left(\sqrt{T}+T/n\right)\log^{2}n,\frac{M}{\sqrt{n}}\right\}.$$
*(2)*  *If in addition the loss is also smooth (cf. Assumption 5), we have*
$$\begin{array}{c} E_{\phi ∼Q}\left[G(S,\phi )\right]≲\left(\;T\log n\;-\;H\left(Q\right)\;+\;\log\frac{1}{\delta }\;\right)\\ \max\left\{L^{2}\eta \exp\left(\alpha^{2}t\eta^{2}\right)\left(\frac{T}{n}+1+\sqrt{\frac{T}{n}}\right)\log^{2}n,\frac{M}{\sqrt{n}}\right\}. \end{array}$$

Let us assume again that $\mathrm{KL}(Q∥U)\in \tilde{O}\left(1\right)$. As Theorem 2 deals with min-max optimization applicable to minimizing an adversarially robust loss function, this assumption still allows some extra flexibility to account for a few outliers while having the following rates. For part 1, if we choose $T=O(n^{2})$ and $\eta =O\left(T^{-3/4}\right)$, this gives a rate of the order $\tilde{O}\left(1/\sqrt{n}\right)$. For part 2, if we choose T=O(n) and $\eta =O(1/\sqrt{n})$, this gives the bounds of the order $\tilde{O}\left(1/\sqrt{n}\right)$.

## 5. Algorithmic Implications and Illustrative Experiments

Our theoretical guarantees in the previous section are given up to constant factors. This is common in theoretical analyses, as such results still give useful information about the behavior of bounds with quantities of interest, such as the sample size *n*. To further verify that our bounds are informative, in this section we show how one can convert them into learning algorithms by minimizing the terms on the r.h.s. of our bounds. We will then illustrate the working of the resulting algorithms in numerical experiments and demonstrate examples of extracting meaningful information from these new algorithms. The goal of this section is to empirically corroborate our theoretical guarantees and demonstrate their potential use for algorithm design.

In line with our theory, we use uniform sampling as the PAC–Bayes prior, and we learn the posterior sampling along with the model’s parameters, by alternating minimization of our bounds. We choose $Q=q^{T}$ of a factorized form, which corresponds to sampling from training indices [n] with replacement *T* times during the training trajectory. Minimizing the terms on the r.h.s. of the bounds in Theorem 1 yields an adaptive Pairwise SGD algorithm that we refer to as Pairwise SGD-Q, and likewise minimizing the r.h.s. of the bounds in Theorem 2 yields an adaptive Pairwise SGDA algorithm SGDA-Q. The pseudo-codes of both of these resulting algorithms are given in Algorithm 1 (with the options of Pairwise SGD-Q or Pairwise SGDA-Q).

**Algorithm 1** Pairwise SGD-Q/Pairwise SGDA-Q
1:**Inputs:** $\left\{\left(i_{t},j_{t}\right):i_{t},j_{t}\in \left[n\right],i_{t}\neq j_{t}\right\}$, *ℓ*, ν, $T_{iter}$, Epochs2:**Initialize:** q← uniform, $w_{0}=0$, $v_{0}=0$, t←13:**for** epoch=1toEpochs **do**4:   **for** $t=1\;\mathrm{to}\;T_{iter}$ **do**5:     Sample $(i_{t},j_{t})∼q$6:     **Pairwise SGD-Q:**7:     $\;\;w_{t+1}=w_{t}-\eta \nabla_{w}\ell \left(w_{t};z_{i_{t}},z_{j_{t}}\right)$;8:     **Pairwise SGDA-Q:**9:     $\;\;v_{t+1}=v_{t}+\eta \nabla_{v}\ell \left(\left(w_{t},v_{t}\right);z_{i_{t}},z_{j_{t}}\right)$;10:     $\;\;w_{t+1}=w_{t}-\eta \nabla_{w}\ell \left(\left(w_{t},v_{t}\right);z_{i_{t}},z_{j_{t}}\right)$;11:     Let t←t+112:   **end for**13:   Update all *q* as $q\left(i,j\right)\propto \exp\left(-\frac{1}{\nu }\ell \left(w;z_{i},z_{j}\right)\right)$ for each pair14:
**end for**
15:**Return** w, v, *q*


Before moving on to exemplify our algorithms at work, we should note that there are two in-built guardrails that prevent sampling bias by design, as follows. The r.h.s. of all bounds that we minimize contain two key terms, each acting as a guardrail: (i) Minimizing the $D_{\mathrm{KL}(Q∥P)}$ term is equivalent to maximizing the entropy of *Q*, i.e., the sampling distribution that we learn (since *P* is the uniform sampling)—hence, *Q* will only deviate from uniformity for a good reason (e.g., when encountering misleading or mislabeled training pairs). (ii) Minimizing the *expected* empirical risk (rather than the empirical risk itself) has the consequence that the correction term that usually appears in existing non-uniform sampling-based SGD-type algorithms simply cancels out, automatically ensuring unbiased gradients by construction and without an external correction. Overall, this illustrates the advantage of learning algorithms obtained by minimizing generalization bounds, as the two terms together minimize an upper bound on the quantity we care about, i.e., the true risk.

The forthcoming numerical experiments are meant to showcase the way in which our adaptive sampling methods enhance robustness by learning to down-weight misleading or mislabeled training examples, thereby avoiding being misled on them. As a byproduct, these weights provide explanatory information about the data pairs.

### Numerical Results in Pairwise Preference Learning

We illustrate the working of our bound-based pairwise learning algorithms on a toy problem involving pairwise ranking on synthetic 2D data. Pairwise ranking is the task of inferring relative preferences by comparing items in pairs. It has broad applicability, including information retrieval, recommendation systems, preference modeling [53], and positive-unlabeled learning [12]. In this section, we present experiments using a simple linear preference model to demonstrate how our theoretical findings manifest in practice. The aim of this section is not to compete with state-of-the-art empirical methods, but rather to provide insight into the behavior of the algorithms and the use of our bounds under controlled conditions.

We generate n=50 i.i.d. points from a 2D standard Gaussian and assign “preference scores” to each using a hidden linear function $s_{true}\left(x_{i}\right)=w_{true}^{T}x_{i},i\in \left[n\right]$, where $w_{\mathrm{true}}={\left(1.5,-1\right)}^{T}$ is fixed. We then sample 1000 pairs from this data and assign binary labels $1(w_{true}x_{i}\leq w_{true}x_{j})$ indicating which of two items is preferred in each sampled pair.

We use the resulting labeled pairs in the form of difference vectors $x_{i}-x_{j}$ (where i,j∈[n],i≠j) as inputs to our Pairwise SGD-Q algorithm to train a linear model with cross-entropy loss for 15 epochs of $T_{iter}$ iterations, each set equal to the number of pairs (so $T=T_{iter}\cdot Epochs$), with step size η=0.1. We aim to learn a scoring function so that for any two items $x_{i}$ and $x_{j}$ the model can say which one ranks higher. The model learns a weight vector w so that the score of item *x* is $s\left(x\right)=w^{T}x$, and we want $w^{T}x_{i}>w^{T}x_{j}$ if item *i* is preferred to *j*. That is, the model projects all points onto the direction w and ranks them by how far they fall along that line.

**Figure 1:**
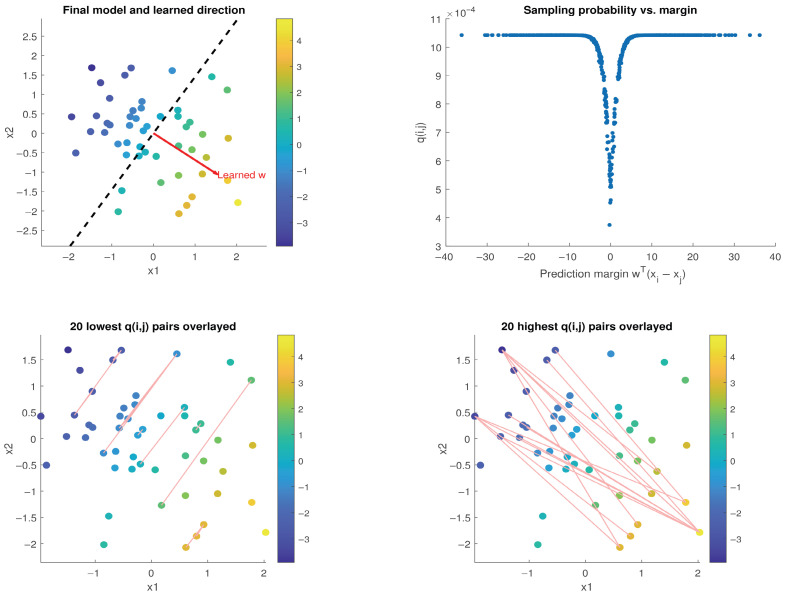
Visualization of Pairwise SGD-Q on a synthetic pairwise ranking task with 2D Gaussian data. (**Top-Left**) Data points colored by true preference scores; the red arrow indicates the learned ranking direction, and the dashed line shows the decision boundary $w^{⊤}x=0$. (**Top-Right**) Pairwise margins $w^{⊤}\left(x_{i}-x_{j}\right)$ vs. sampling probabilities q(i,j); high-confidence pairs (larger margins) are sampled more frequently. (**Bottom-Left**) The 20 least informative (lowest *q*) pairs—these are distant pairs having similar preference scores. (**Bottom-Right**) The 20 most informative (highest *q*) pairs.

The results are plotted in Figure 1. The top-left figure shows the 2D data colored by their preference scores. A red arrow shows the ranking direction learned by our Pairwise SGD-Q, i.e., the direction the trained model uses to order points. Its associated “decision boundary” is shown by the dashed line. In this context, the decision boundary represents the level set $w^{T}x=0$ that is the dividing line (more generally, hyperplane) orthogonal to the learned ranking direction. It shows how w splits the space into higher- and lower-scoring regions.

The top-right figure is a scatter plot showing the relationship between the learned pairwise margins $w^{T}\left(x_{i}-x_{j}\right)$ and the learned sampling probabilities q(i,j) (q-scores for each pair). Ambiguous pairs with small margins, i.e., those the model is less confident about, tend to have higher losses and thus get down-weighted by a lower sampling probability q(i,j). Hence, more confident (larger-margin) pairs are sampled more often.

The bottom-left plot overlays an edge for the 20 lowest *q*-scored pairs. These are the pairs that have the same preference score but differing feature coordinates—indeed the least helpful pairs for learning the preference change direction. The 20 highest q-scored pairs are shown on the bottom-right plot; these are most informative of the direction of change.

**Figure 2:**
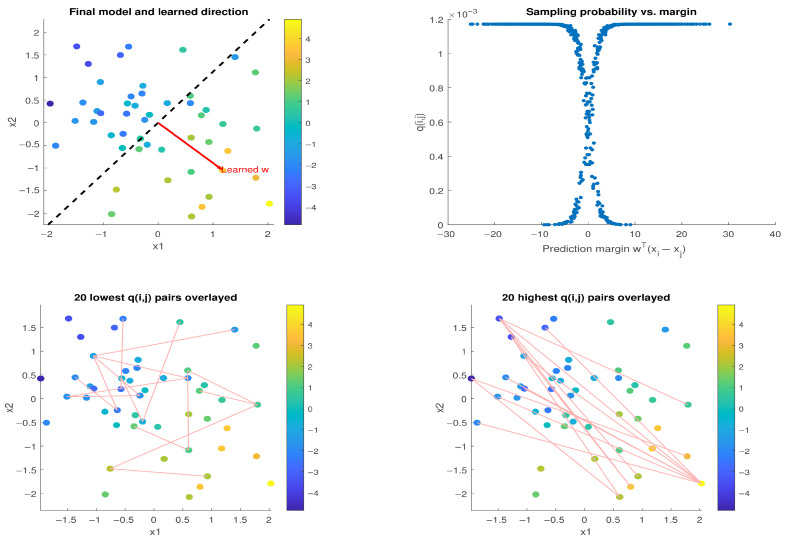
Same setup as Figure 1, but with additive Gaussian noise in the generating preference model. The top-right plot shows that q(i,j) now reflect both the size of pairwise margin and the noise sensitivity, assigning lower weights to mislabeled or ambiguous pairs. The bottom-left plot shows that, as before, Pairwise SGD-Q still down-weights distant pairs that have too similar preference scores, although now these are no longer perpendicular to the ranking direction due to noise. The bottom-right plot shows that Pairwise SGD-Q prioritizes the pairs most aligned with the ranking direction.

We repeated the experiment using a noisy preference model, where the observed scores are given by $s_{true}\left(x_{i}\right)=w_{true}^{⊤}x_{i}+\upsilon_{i}$, with $\upsilon_{i}{∼}_{i.i.d.}N\left(0,1\right)$ for i∈[n]. Figure 2 shows the results of training a model of the same form as previously, using our Pairwise SGD-Q. The top-left plot depicts the noisy data overlaid with the learned direction w. The top-right plot shows the pairwise margins $w^{⊤}\left(x_{i}-x_{j}\right)$ against the corresponding sampling probabilities q(i,j). While *q* still decreases near the pairwise margin, the noise now causes mislabeled or ambiguous pairs to receive even lower *q* values. This becomes evident in the bottom-left plot: the lowest *q*-scored pairs have similar preference scores but differing features and are no longer orthogonal to w due to noise. The bottom-right plot shows the highest *q*-scored pairs, which continue to reflect the most informative preference differences aligned on average with the learned direction.

Next, we plot learning curves to see how the generalization performance of the pairwise ranking model trained with our Pairwise SGD-Q and Pairwise SGDA-Q algorithms varies with the number of i.i.d. items, under both clean and noisy settings, while we keep the number of pairs used for training constant. The Pairwise SGDA-Q experiments represent an instance of adversarial training. This can model, for instance, malicious users, bots, or strategic agents in applications like recommendation systems, crowd-sourced ranking, sports, or election ranking.

We vary n∈{5,10,20,30,40,50,60,70,80,90,100} and repeat all experiments on both clean and noisy preference score generation models. This totals 44 different experiment settings, and we perform 50 independent trials for each. The data generation process is the same as before. While the sample size *n* varies, the number of pairs sampled from these *n* points for training is set to 1000 throughout, and these pairs are labeled in the same way as before.

Training with Pairwise SGD-Q is performed in the same way as previously described, but this time we set $T_{iter}=300$ to run for 30 epochs. When training with Pairwise SGDA-Q, at each training epoch the algorithm first computes the pairwise losses after adversarial maximization over weight vector v constrained to an $\ell_{2}$-ball of radius ϵ=0.05 around the current model w, using 6 gradient ascent steps with step size η=0.1. The resulting adversarially induced losses are used to compute the sampling distribution $q\left(i,j\right)\propto \exp\left(-\ell_{ij}\right)$, from which $T_{iter}=300$ pairs are drawn to iteratively update w, for 30 epochs.

For both algorithms, evaluation is performed using an independent test set of 500 unseen items, drawn from the clean preference scoring model. For Pairwise SGD-Q, the out-of-sample errors are computed. For Pairwise SGDA-Q, three different out-of-sample error metrics are computed: (1) standard pairwise error, (2) error under adversarial perturbation of the model’s weights w, and (3) error under adversarial perturbation of the test pairwise inputs $x_{i}-x_{j}$, all within the same $\ell_{2}$ radius and using the same Pairwise SGDA-Q ascent procedure.

**Figure 3:**
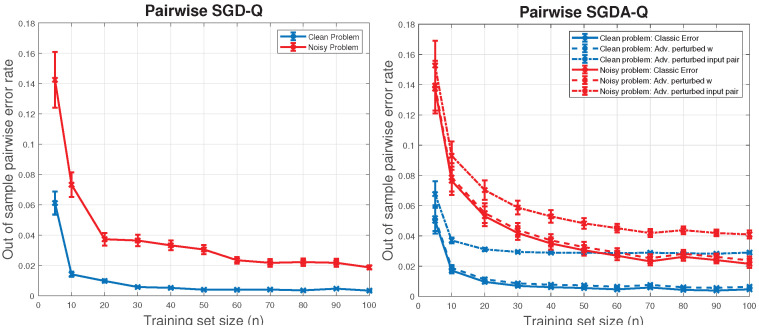
Out-of-sample errors for Pairwise SGD-Q and Pairwise SGDA-Q as a function of the number of i.i.d. items *n*, while the number of pairs trained on remains constant at 1000. The curves represent averages, and the error bars are standard errors from 50 independent trials.

Figure 3 reports the obtained learning curves for both algorithms: the error bars show the average and standard error across 50 independent runs. From these figures we can see, as expected, that both natural noise and adversarial perturbations make the problem harder. However, all out-of-sample errors display a decreasing trajectory as the sample size grows.

## 6. Conclusions

We obtained stability-based PAC–Bayes bounds for randomized pairwise learning, applicable to general sampling. These bounds are applicable to analyzing the generalization of stochastic optimization algorithms, and we demonstrated this in the case of Pairwise SGD and Pairwise SGDA. Our generalization analysis of these methods is suggestive of new stochastic optimizers that allow non-uniform and data-dependent sampling distributions to be updated during the training process. We believe this is a theoretically grounded step that connects two important ideas and may support future work on more complex or application-specific methods. The practical use of this idea is explored in a companion paper [22].

Limitations: Our analysis of Pairwise SGD and Pairwise SGDA is built on a set of classic assumptions regarding the loss function, with convexity being perhaps the most restrictive among them. Nonetheless, insights gained from the convex setting remain a valuable stepping stone to tackling more general, non-convex problems in future work. Indeed, a worthwhile avenue will be to obtain bounds and associated algorithms under relaxed assumptions. Furthermore, here we demonstrated numerical results with our bound-based algorithm under its intended conditions. It will also be interesting to explore experimentally to what extent such algorithms remain functional and potentially useful outside the theoretical conditions in which they were obtained.

## Figures and Tables

**Table 1:** Summary of generalization rates obtained for two pairwise stochastic optimization algorithms (Pairwise SGD, Pairwise SGDA) under two sets of assumptions (Lipschitz (L), smooth (S), convex (C)) on the pairwise loss function, together with the chosen number of iterations *T* and step size η. The sample size is *n*. According to this summary, we notice that smaller step sizes and more iterations are needed if the smoothness assumption is removed (more details in Section 4).

Algo.	Asm.	Time *T* and Step Size η		Rates
Pairwise SGD	L, C	$T=\Theta (n^{2})$	$\eta =\Theta (T^{-\frac{3}{4}})$	$\tilde{O}\left(1/\sqrt{n}\right)$
				Theorem 1 (1)
	L, S, C	T=Θ(n)	$\eta =\Theta (T^{-\frac{1}{2}})$	$\tilde{O}\left(1/\sqrt{n}\right)$
				Theorem 1 (2)
Pairwise SGDA	L, C	$T=O\left(n^{2}\right)$	$\eta =O(T^{-\frac{3}{4}})$	$\tilde{O}\left(1/\sqrt{n}\right)$
				Theorem 2 (1)
	L, S, C	T=O(n)	$\eta =O(T^{-\frac{1}{2}})$	$\tilde{O}\left(1/\sqrt{n}\right)$
				Theorem 2 (2)

## Data Availability

The data presented in this study are available on request from the corresponding author.

## Appendix A. Proof of Lemma 1

We follow the ideas in [31,54] to prove Lemma 1. We first introduce some useful lemmas. The following lemma shows some results on characterizing sub-Gaussian and sub-exponential random variables. For λ>0, let E[exp(λZ)] denote the moment-generating function (MGF) of *Z*. We denote I[·] the indicator function.

**Lemma** **A1** (Vershynin [55]). *Let X be a random variable with E[X]=0. We have the following equivalences for X:*

- *${∥X∥}_{p}={\left(E|X\right|}^{p}{)}^{1/p}\leq \sqrt{p}$, for all p≥1.*
- *There exists $K_{1}\geq 0$ such that, for all λ∈R, $E\left[\exp\left(\lambda X\right)\right]\leq \exp\left(K_{1}\lambda^{2}\right).$*

*We have the following equivalences for X:*

- *${∥X∥}_{p}={\left(E|X\right|}^{p}{)}^{1/p}\leq p$, for all p≥1.*
- *For all λ such that $\left|\lambda \right|\leq \frac{1}{2e}$, $E\left[\exp\left(\lambda X\right)\right]\leq \exp\left(2e^{2}\lambda^{2}\right).$*

The following lemma gives a change in measure of the KL divergence.

**Lemma** **A2** (Lemma 4.10 in Van Handel [56]). *For any measurable function g:Φ↦R, we have*

$$\log E_{\phi ∼P}\left[\exp\left(g\left(\phi \right)\right)\right]=\underset{Q}{\sup}\left[E_{\phi ∼Q}\left[g\left(\phi \right)\right]-\mathrm{KL}\left(Q∥P\right)\right].$$

We denote the $L_{p}$-norm of a random variable *Z* as ${∥Z∥}_{p}:={\left(E[|Z{|}^{p}]\right)}^{\frac{1}{p}},p\geq 1$, denote $S\setminus \{z_{i}\}$ the set $\left\{z_{1},\ldots ,z_{i-1},z_{i+1},\ldots ,z_{n}\right\}$, and abbreviate $\sum_{i,j\in [n]:i\neq j}$ as $\sum_{i\neq j}$. For $z_{k}^{\prime }\in Z$, $S^{\left(k\right)}$ is the set derived by replacing the *k*-th element of *S* with $z_{k}^{\prime }$.

The following lemma gives moment bounds for a summation of weakly dependent and mean-zero random functions with bounded increments under a small change.

**Lemma** **A3** (Theorem 1 in Lei et al. [33]). *Let $S=\{z_{1},\ldots ,z_{n}\}$ be a set of independent random variables that each takes values in Z and M>0. Let $g_{i,j},\forall i,j\in \left[n\right],i\neq j$ be some functions that can be decomposed as $g_{i,j}=g_{j}^{\left(i\right)}+{\tilde{g}}_{i}^{\left(j\right)}$. Suppose for $g_{j}^{\left(i\right)}:Z^{n}\mapsto R$ and ${\tilde{g}}_{i}^{\left(j\right)}:Z^{n}\mapsto R$, the following hold for any i,j∈[n],i≠j:*

- *$|E_{S\setminus \{z_{j}\}}\left[g_{j}^{\left(i\right)}\left(S\right)\right]|\leq 2M,\;\mathrm{and}\;|E_{S\setminus \{z_{i}\}}\left[{\tilde{g}}_{i}^{\left(j\right)}\left(S\right)\right]|\leq 2M$ almost surely (a.s.).*
- *$E_{z_{j}}\left[g_{j}^{\left(i\right)}\left(S\right)\right]=0,\;\mathrm{and}\;E_{z_{i}}\left[{\tilde{g}}_{i}^{\left(j\right)}\left(S\right)\right]=0$ a.s.*
- *For any j∈[n] with i≠j,k≠j we have $|g_{j}^{\left(i\right)}\left(S\right)-g_{j}^{\left(i\right)}\left(S^{\left(k\right)}\right)|\leq 2\beta$ a.s., and for any i∈[n] with j≠i and k≠i, we have $|{\tilde{g}}_{i}^{\left(j\right)}\left(S\right)-{\tilde{g}}_{i}^{\left(j\right)}\left(S^{\left(k\right)}\right)|\leq 2\beta$ a.s.*

*Then, we can decompose $\sum_{i\neq j}g_{j}^{\left(i\right)}\left(S\right)$ and $\sum_{i\neq j}{\tilde{g}}_{i}^{\left(j\right)}\left(S\right)$ as follows:*

$$\sum_{i\neq j}g_{j}^{\left(i\right)}\left(S\right)=X_{1}+X_{2},\;\mathrm{and}\;\sum_{i\neq j}{\tilde{g}}_{i}^{\left(j\right)}\left(S\right)={\tilde{X}}_{1}+{\tilde{X}}_{2}$$

*where $X_{1}$, $X_{2}$, ${\tilde{X}}_{1}$, ${\tilde{X}}_{2}$ are four random variables satisfying $E\left[X_{1}\right]=E\left[X_{2}\right]=E\left[{\tilde{X}}_{1}\right]=E\left[{\tilde{X}}_{2}\right]=0$. Furthermore, for any p≥1*

$$∥X_{1}{∥}_{p}\leq 8M\sqrt{p(n-1)}n\;\mathrm{and}\;{∥{\tilde{X}}_{1}∥}_{p}\leq 8M\sqrt{p(n-1)}n$$

*and for any p≥2*

$$∥X_{2}{∥}_{p}\leq 24\sqrt{2}p\left(n-1\right)n\beta \left\lceil \log_{2}\left(n-1\right)\right\rceil \;\mathrm{and}\;{∥{\tilde{X}}_{2}∥}_{p}\leq 24\sqrt{2}p\left(n-1\right)n\beta \left\lceil \log_{2}\left(n-1\right)\right\rceil .$$

**Proof** **of Lemma 1.** Based on Lemma A2, if we set g(ϕ)=λh(ϕ), then

(A1) $$E_{Q}\left[h\left(\phi \right)\right]\leq \frac{1}{\lambda }\left(\log E_{P}\left[\exp\left(\lambda h\left(\phi \right)\right)\right]+\mathrm{KL}\left(Q∥P\right)\right).$$

To control the deviations of $\log E_{P}\left[\exp\left(\lambda h\left(\phi \right)\right)\right]$, we use Markov’s inequality. With a probability of 1−ϵ, we have

$$E_{P}\left[e^{\lambda h(\phi )}\right]\leq \frac{E_{S}E_{P}\left[e^{\lambda h(\phi )}\right]}{\epsilon }.$$

Applying the above results to Equation (A1), with a probability of 1−ϵ, we get

(A2) $$E_{Q}\left[h\left(\phi \right)\right]\leq \frac{1}{\lambda }\left(\log E_{P}\left[e^{\lambda h(\phi )}\right]+\mathrm{KL}\left(Q∥P\right)\right)\leq \frac{1}{\lambda }\left(\log\frac{E_{S}E_{P}\left[e^{\lambda h(\phi )}\right]}{\epsilon }+\mathrm{KL}\left(Q∥P\right)\right).$$

We can exchange $E_{P}$ and $E_{S}$ using Fubini’s theorem. Next, we will bound the generalization gap with respect to P. Let δ be some decaying function of *n*. We denote by $\Omega_{\delta }$ a subset with $Pr(\Omega_{\delta })\geq 1-\delta$ on which sub-exponential stability (Definition 2) holds and $\Omega_{\delta }^{c}$ is the complement of $\Omega_{\delta }$. We first give results for any fixed $\phi \in \Omega_{\delta }$. Given $\phi \in \Omega_{\delta }$, it was shown in Lei et al. [33], ∀i,j∈[n], that

$$G\left(S,\phi \right)\leq 4\beta_{\phi }+\frac{1}{n(n-1)}\sum_{i\neq j}g_{i,j}\left(S\right),$$

$$g_{i,j}\left(S\right)=E_{z_{i}^{\prime },z_{j}^{\prime }}\left[E_{Z,\tilde{Z}}\left[\ell \left(A\left(S_{i,j}\right);Z,\tilde{Z}\right)\right]-\ell \left(A\left(S_{i,j}\right);z_{i},z_{j}\right)\right].$$

As shown in Lei et al. [33], $g_{i,j}$ satisfies all the conditions in Lemma A3, and therefore one can apply Lemma A3 to show the existence of four random variables $X_{1}$, $X_{2}$, ${\tilde{X}}_{1}$, ${\tilde{X}}_{2}$ such that $E\left[X_{1}\right]=E\left[X_{2}\right]=E\left[{\tilde{X}}_{1}\right]=E\left[{\tilde{X}}_{2}\right]=0$:

$$\frac{1}{n(n-1)}\sum_{i\neq j}g_{i,j}\left(S\right)=X_{1}+X_{2}+{\tilde{X}}_{1}+{\tilde{X}}_{2}$$

$$\begin{array}{c} \mathrm{and}\;∥X_{1}{∥}_{p}\leq 8\sqrt{p}M{\left(n-1\right)}^{-\frac{1}{2}},\;\forall p\geq 1,\;{∥{\tilde{X}}_{1}∥}_{p}\leq 8\sqrt{p}M{\left(n-1\right)}^{-\frac{1}{2}},\;\forall p\geq 1,\\ ∥X_{2}{∥}_{p}\leq 24\sqrt{2}p\beta_{\phi }\left\lceil \log_{2}\left(n-1\right)\right\rceil ,\;\forall p\geq 2,\;{∥{\tilde{X}}_{2}∥}_{p}\leq 24\sqrt{2}p\beta_{\phi }\left\lceil \log_{2}\left(n-1\right)\right\rceil ,\;\forall p\geq 2. \end{array}$$

Using the first part of Lemma A1 with $X=X_{1}/8M{\left(n-1\right)}^{-\frac{1}{2}}$ to get

(A3) $$\max\left\{E_{S}\left[\exp\left(\lambda X_{1}\right)\right],E_{S}\left[\exp\left(\lambda {\tilde{X}}_{1}\right)\right]\right\}\leq \exp\left(64M^{2}{\left(n-1\right)}^{-1}K_{1}\lambda^{2}\right)$$

and using the second part of Lemma A1 with $X=X_{2}/24\sqrt{2}\beta_{\phi }\left\lceil \log_{2}\left(n-1\right)\right\rceil$,

(A4) $$\begin{array}{c} \max\left\{E_{S}\left[\exp\left(\lambda X_{2}\right)\right],E_{S}\left[\exp\left(\lambda {\tilde{X}}_{2}\right)\right]\right\}\leq \exp\left[2304e^{2}\beta_{\phi }^{2}{\left\lceil \log_{2}\left(n-1\right)\right\rceil }^{2}\lambda^{2}\right],\\ \forall |\lambda |\leq \frac{1}{48e\sqrt{2}\beta_{\phi }\left\lceil \log_{2}\left(n-1\right)\right\rceil }. \end{array}$$

According to Jensen’s inequality, we have

$$\begin{array}{c} \exp\left(\lambda X_{1}+\lambda X_{2}+\lambda {\tilde{X}}_{1}+\lambda {\tilde{X}}_{2}\right)=\exp\left(\lambda X_{1}\right)\exp\left(\lambda X_{2}\right)\exp\left(\lambda {\tilde{X}}_{1}\right)\exp\left(\lambda {\tilde{X}}_{2}\right)\\ \leq \frac{1}{4}\left(\exp\left(4\lambda X_{1}\right)+\exp\left(4\lambda X_{2}\right)+\exp\left(4\lambda {\tilde{X}}_{1}\right)+\exp\left(4\lambda {\tilde{X}}_{2}\right)\right). \end{array}$$

This implies

$$\begin{array}{cc} \; & E_{S}\exp\left[\lambda G\left(S,\phi \right)\right]\leq E_{S}\exp\left[\lambda \left(4\beta_{\phi }+X_{1}+X_{2}+{\tilde{X}}_{1}+{\tilde{X}}_{2}\right)\right]\\ \; & \leq \exp\left(4\lambda \beta_{\phi }\right)\frac{1}{4}\left(E_{S}\left[\exp\left(4\lambda X_{1}\right)+\exp\left(4\lambda X_{2}\right)+\exp\left(4\lambda {\tilde{X}}_{1}\right)+\exp\left(4\lambda {\tilde{X}}_{2}\right)\right]\right). \end{array}$$

As sub-exponential stability (Definition 2) holds with $\beta_{\phi }\leq c_{1}+c_{2}\log\left(1/\delta \right)$ when $\phi \in \Omega_{\delta }$, the above inequality together with Equations (A3) and (A4) implies that, for all

$$0<\lambda \leq \frac{1}{192e\sqrt{2}\left(c_{1}+c\log\left(1/\delta \right)\right)\left\lceil \log_{2}\left(n-1\right)\right\rceil },$$

we have

(A5) $$\begin{array}{c} E_{S}\left[\exp\left(\lambda G\left(S,\phi \right)\right)\right]\leq \exp\left(4\lambda \left(c_{1}+c\log\left(1/\delta \right)\right)\right)(\exp\left(256M^{2}{\left(n-1\right)}^{-1}K_{1}\lambda^{2}\right)\\ +\exp(9216\times {\left(2e\right)}^{2}{\left(c_{1}+c\log\left(1/\delta \right)\right)}^{2}{\left\lceil \log_{2}\left(n-1\right)\right\rceil }^{2}\lambda^{2})). \end{array}$$

Next, we give results for any fixed ϕ. We define $H:Z^{n}\times \Phi \mapsto R$ as $H\left(S,\phi \right)=G\left(S,\phi \right)I\left[\phi \in \Omega_{\delta }\right]$, where I[·] is the indicator function. We have

(A6) $$E_{Q}\left[G\left(S,\phi \right)\right]=E_{Q}\left[H\left(S,\phi \right)\right]+E_{Q}\left[G\left(S,\phi \right)|\phi \in \Omega_{\delta }^{c}\right]Q\left(\Omega_{\delta }^{c}\right).$$

Based on Equation (A.8) and Equation (A.9) in Zhou et al. [31], for α>1, we have

(A7) $$E_{Q}\left[G\left(S,\phi \right)\right]\leq E_{Q}\left[H\left(S,\phi \right)\right]+M\underset{\alpha >1}{\inf}\delta^{\frac{\alpha -1}{\alpha }}{\left(E_{P}\left[{\left(\frac{Q(\phi )}{P(\phi )}\right)}^{\alpha }\right]\right)}^{\frac{1}{\alpha }},$$

where ℓ(A(S;ϕ))∈[0,M] and

(A8) $$\begin{array}{c} E_{S}E_{P}\left[\exp\left(\lambda H\left(S,\phi \right)\right)\right]\leq E_{S}E_{P}\left[\exp\left(\lambda \left(G(S,\phi )\right)|\phi \in \Omega_{\delta }\right)\right]+\delta . \end{array}$$

Combining the above Equation (A8) with Equation (A5), we obtain

(A9) $$\begin{array}{c} E_{P}E_{S}\left[\exp\left(\lambda H\left(S,\phi \right)\right)\right]\leq \exp\left(2\lambda \left(c_{1}+c\log\left(1/\delta \right)\right)\right)\times \left(\exp(256M^{2}{\left(n-1\right)}^{-1}K_{1}\lambda^{2})+\right.\\ \left.\exp(9216\times {\left(2e\right)}^{2}{\left(c_{1}+c_{2}\log\left(1/\delta \right)\right)}^{2}{\left\lceil \log_{2}\left(n-1\right)\right\rceil }^{2}\lambda^{2})\right)+\delta . \end{array}$$

For any u,v,w>0 and δ∈(0,1), we have

exp(u)(exp(v)+exp(w))+δ≤exp(u+1/2)(exp(v)+exp(w)).

Applying the above inequality into Equation (A9), if $u=2\lambda (c_{1}+c_{2}\log\left(1/\delta \right))$, $v=256M^{2}n^{-1}K_{1}\lambda^{2}$, $w=9216\times {\left(2e\right)}^{2}{\left(c_{1}+c_{2}\log\left(1/\delta \right)\right)}^{2}{\left\lceil \log_{2}n\right\rceil }^{2}\lambda^{2}$, it gives

(A10) $$\begin{array}{c} E_{P}E_{S}\left[\exp\left(\lambda H\left(S,\phi \right)\right)\right]\leq \exp\left(2\lambda (c_{1}+b\log\left(1/\delta \right))+1/2\right)\times \left(\exp(256M^{2}\frac{K_{1}\lambda^{2}}{n-1})+\right.\\ \left.\exp(9216\times {\left(2e\right)}^{2}{\left(c_{1}+c_{2}\log\left(\frac{1}{\delta }\right)\right)}^{2}{\left\lceil \log_{2}\left(n-1\right)\right\rceil }^{2}\lambda^{2})\right). \end{array}$$

We choose

(A11) $$\lambda =\min\left\{\frac{1}{192e\sqrt{2}\left(c_{1}+c_{2}\log\left(1/\delta \right)\right)\left\lceil \log_{2}\left(n-1\right)\right\rceil },\frac{\sqrt{\left(n-1\right)}}{16\sqrt{K_{1}}M}\right\},$$

so that we have

$$\begin{array}{c} 2\lambda (c_{1}+c_{2}\log\left(1/\delta \right))+1/2\leq 1,\\ 256M^{2}{\left(n-1\right)}^{-1}K_{1}\lambda^{2}\leq 1,\\ 9216\times {\left(2e\right)}^{2}{\left(c_{1}+c_{2}\log\left(1/\delta \right)\right)}^{2}{\left\lceil \log_{2}\left(n-1\right)\right\rceil }^{2}\lambda^{2}\leq 1. \end{array}$$

Plugging this back into Equation (A10) yields the MGF of our truncated generalization gap, H(S;ϕ), which is a key quantity in PAC–Bayes analysis:

$$E_{P}E_{S}\left[\exp\left(\lambda H\left(S,\phi \right)\right)\right]\leq e\left(e+e\right)\leq e^{3}.$$

Applying the above results into Equation (A2), we have, with a probability of $1-\delta^{\prime }$,

$$E_{Q}\left[H\left(S,\phi \right)\right]\leq \frac{1}{\lambda }\left(\log\left(e^{3}/\delta^{\prime }\right)+\mathrm{KL}\left(Q∥P\right)\right)=\frac{1}{\lambda }\left(3+\log\left(1/\delta^{\prime }\right)+\mathrm{KL}\left(Q∥P\right)\right).$$

Based on the above inequality and Equations (A7) and (A8), the following inequality holds uniformly for all Q with probability of at least $1-\delta^{\prime }$:

$$\begin{array}{cc} \; & E_{\phi ∼Q}\left[G\left(S,\phi \right)\right]\leq E_{\phi ∼Q}\left[H\left(S,\phi \right)\right]+M\underset{\alpha >1}{\inf}\delta^{\frac{\alpha -1}{\alpha }}{\left(E_{P}\left[{\left(\frac{Q(\phi )}{P(\phi )}\right)}^{\alpha }\right]\right)}^{\frac{1}{\alpha }}\\ & \leq \frac{\mathrm{KL}\left(Q∥P\right)+\log\left(1/\delta^{\prime }\right)+3}{\lambda }+M\underset{\alpha >1}{\inf}\delta^{\frac{\alpha -1}{\alpha }}{\left(E_{P}\left[{\left(\frac{Q(\phi )}{P(\phi )}\right)}^{\alpha }\right]\right)}^{\frac{1}{\alpha }}. \end{array}$$

In the above, comparing the first and the second term on the r.h.s, the second term can be made negligible by choosing δ small enough, $\delta \ll n^{-T}$. Therefore, our analysis shows

$$E_{\phi ∼Q}\left[G(S,\phi )\right]≲\left(\mathrm{KL}\left(Q∥P\right)+\log\left(1/\delta_{1}\right)\right)\max\left\{\left(c_{1}+c_{2}\log\left(n\right)\right)\left\lceil \log_{2}n\right\rceil ,\frac{M}{\sqrt{n}}\right\}.$$

The proof is completed. □

Here, we discuss the existence of $E_{\phi ∼P}\left[{\left(\frac{Q(\phi )}{P(\phi )}\right)}^{\alpha }\right]$. In practice, we consider Q and P to be sampling distributions. In these cases, Q and P are discrete distributions on the same dataset. In particular, we are interested in the case with P being the uniform distribution. Under these circumstances, this expectation exists.

## Appendix B. Proofs for Pairwise SGD and Pairwise SGDA with Adaptive Sampling

### Appendix B.1. Pairwise Stochastic Gradient Descent

We will prove that stability bounds of Pairwise SGD satisfy sub-exponential stability (Definition 2). Based on this, we can derive the generalization bounds for Pairwise SGD with smooth and non-smooth convex loss functions. To this aim, we introduce the following lemma to bound the summation of i.i.d events [57].

**Lemma** **A4** (Chernoff’s Bound).

*Let $Z_{1},\ldots ,Z_{t}$ be independent random variables taking values in {0,1}. Let $Z=\sum_{k=1}^{t}Z_{k}$ and μ=E[Z]. Then, for any δ∈(0,1) with probability of at least 1−δ, we have*

$$Z\leq \mu +\log\left(1/\delta \right)+\sqrt{2\mu \log(1/\delta )}.$$

We first present the stability bounds for non-smooth and convex cases.

**Proof** **of Lemma 2, (1).** Without loss of generality, we assume *S* and $S^{\prime }$ differ by the last example. Based on the Equation (F.2) in Lei et al. [51], we have

$$\begin{array}{cc} ∥w_{t+1}-w_{t+1}^{\prime }{∥}_{2}^{2} & \leq 4L^{2}\eta^{2}{\left(1+p\right)}^{\sum_{k=1}^{t}I\left[i_{k}=n\;\;\mathrm{or}\;\;j_{k}=n\right]}\left(t+p^{-1}\sum_{k=1}^{t}I\left[i_{k}=n\;\;\mathrm{or}\;\;j_{k}=n\right]\right). \end{array}$$

We set $p=1/\sum_{k=1}^{t}I\left[i_{k}=n\;\;\mathrm{or}\;\;j_{k}=n\right]$ and use the inequality ${\left(1+1/x\right)}^{x}\leq e$ to get

$$∥w_{t+1}-w_{t+1}^{\prime }{∥}_{2}^{2}\leq 4eL^{2}\eta^{2}\left(t+{\left(\sum_{k=1}^{t}I\left[i_{k}=n\;\;\mathrm{or}\;\;j_{k}=n\right]\right)}^{2}\right).$$

It then follows that

$$∥w_{t+1}-w_{t+1}^{\prime }{∥}_{2}\leq 2\sqrt{e}L\eta \left(\sqrt{t}+\sum_{k=1}^{t}I\left[i_{k}=n\;\;\mathrm{or}\;\;j_{k}=n\right]\right).$$

According to the Lipschitz continuity, we know that Pairwise SGD is $\beta_{\phi }$-uniformly stable with

(A12) $$\beta_{\phi }=2\sqrt{e}L^{2}\eta \left(\sqrt{t}+\max_{k\in [n]}\sum_{m=1}^{t}I\left[i_{m}=k\;\;\mathrm{or}\;\;j_{m}=k\right]\right).$$

To bound $\beta_{\phi }$ w.h.p., we set $\beta_{\phi ,k}=2\sqrt{e}L^{2}\eta \left(\sqrt{t}+\sum_{m=1}^{t}I\left[i_{m}=k\;\;\mathrm{or}\;\;j_{m}=k\right]\right)$ and note that $E\left[I\left[i_{m}=k\;\;\mathrm{or}\;\;j_{m}=k\right]\right]\leq Pr\left\{i_{m}=k\right\}+Pr\left\{j_{m}=k\right\}=2/n.$ Applying Lemma A4 to the sum in Equation (A12), with probability of at least 1−δ/n, we get

$$\begin{array}{c} \beta_{\phi ,k}\leq 2\sqrt{e}L^{2}\eta \left(\sqrt{t}+2t/n+\log\left(n/\delta \right)+2\sqrt{t/n\log(n/\delta )}\right). \end{array}$$

Therefore, with probability of at least 1−δ, the following holds simultaneously for all k∈[n] by the union bound on probability

$$\beta_{\phi ,k}\leq 2\sqrt{e}L^{2}\eta \left(\sqrt{t}+2t/n+\log\left(n/\delta \right)+2\sqrt{t/n\log(n/\delta )}\right).$$

For δ∈(0,1/n), this implies the following inequality with probability of at least 1−δ:

(A13) $$\begin{array}{c} \beta_{\phi }\leq 2\sqrt{e}L^{2}\eta \left(\sqrt{t}+2t/n+2\log\left(1/\delta \right)+2\sqrt{2t/n\log(1/\delta )}\right). \end{array}$$

Finally, from Equation (A13) we know that Pairwise SGD with the uniformly distributed hyperparameter ϕ satisfies sub-exponential stability (Definition 2) with

$$c_{1}=2\sqrt{e}L^{2}\eta \left(\sqrt{t}+2t/n\right),\;c_{2}=4\sqrt{e}L^{2}\eta \left(1+\sqrt{2t/n}\right).$$

The proof is completed. □

**Proof** **of Lemma 2, (2).** By an intermediate result in the proof in Lemma C.3 of Lei et al. [33], for all $z,\tilde{z}\in Z$ and $i_{k},j_{k}\in \left[n\right],i_{k}\neq j_{k}$, with *L*-Lipschitz, we have

$$\left|\ell \left(w_{t+1};z,\tilde{z}\right)-\ell \left(w_{t+1};z,\tilde{z}\right)\right|\leq L{∥w_{t+1}-w_{t+1}^{\prime }∥}_{2}\leq 2L^{2}\sum_{k=1}^{t}\eta_{k}I\left[i_{k}=n\;\mathrm{or}\;j_{k}=n\right].$$

From this inequality it follows that Pairwise SGD is $\beta_{\phi }$-uniformly stable with

(A14) $$\begin{array}{c} \beta_{\phi }=2L^{2}\max_{k\in [n]}\sum_{m=1}^{t}\eta_{m}I\left[i_{m}=k\;\;\mathrm{or}\;\;j_{m}=k\right]. \end{array}$$

Let $\beta_{\phi ,k}=2L^{2}\sum_{m=1}^{t}\eta_{j}I\left[i_{m}=k\;\;\mathrm{or}\;\;j_{m}=k\right]$ for any k∈[n]. It remains to show that the stability parameter of Pairwise SGD satisfies sub-exponential stability (Definition 2). Using Lemma A4 with $Z_{m}=I\left[i_{m}=k\;\;\mathrm{or}\;\;j_{m}=k\right]$ and noting that $E[I\left[i_{m}=k\;\;\mathrm{or}\;\;j_{m}=k\right]]\leq 2/n$, we get the following inequality with probability of at least 1−δ/n (taking $\eta_{j}=\eta$):

(A15) $$\begin{array}{cc} \beta_{\phi ,k}\; & \leq 2L^{2}\eta \left(2t/n+\log\left(n/\delta \right)+2\sqrt{t/n\log(n/\delta )}\right). \end{array}$$

By the union bound, with probability of at least 1−δ, Equation (A15) holds for all k∈[n]. Therefore, with probability of at least 1−δ, it gives

$$\begin{array}{cc} \beta_{\phi } & \leq 2L^{2}\eta \left(2t/n+\log\left(n/\delta \right)+2\sqrt{t/n\log(n/\delta )}\right)\leq 2L^{2}\eta (2t/n+2\log\left(1/\delta \right)+\\ \; & 2\sqrt{2t/n\log(1/\delta )})\leq 4L^{2}\eta t/n+4L^{2}\eta \left(1+\sqrt{2t/n}\right)\log\left(1/\delta \right), \end{array}$$

where we have used δ∈(0,1/n) in the second inequality. Hence, sub-exponential stability (Definition 2) holds with

$$c_{1}=4L^{2}\eta t/n,c_{2}=4L^{2}\eta \left(1+\sqrt{2t/n}\right).$$

This completes the proof. □

**Proof** **of Theorem 1.** With $A\left(S;\phi \right)=w_{T}$, it follows from Lemma 2, (1) and (2), that Pairwise SGD with both convex non-smooth and convex smooth loss functions satisfies sub-exponential stability (Definition 2). Applying the upper bound on $\beta_{\phi }$ to Lemma 1, the result follows. □

### Appendix B.2. Pairwise Stochastic Gradient Descent Ascent

Next, we prove the generalization bounds for Pairwise SGDA with smooth and non-smooth convex-concave loss functions.

**Lemma** **A5** (Lemma C.1., [37]). *Let ℓ be convex-concave.*

(1) *If Assumption 4 holds, then*
  w−η∇wℓ(w,v)v+η∇vℓ(w,v)−w′−η∇wℓ(w′,v′)v′+η∇vℓ(w′,v′)22≤w−w′v−v′22+8L2η2.
(2) *If Assumption 5 holds, then*
  w−η∇wℓ(w,v)v+η∇vℓ(w,v)−w′−η∇wℓw′,v′v′+η∇vℓw′,v′22≤1+α2η2w−w′v−v′22.

**Proof** **of Lemma 3, (1).** We assume *S* and $S^{\prime }$ differ by the last example for simplicity. Based on Lemma A5 (1), for $i_{t}\neq n,j_{t}\neq n,\;\mathrm{and}\;i_{t}\neq j_{t}$, we have

(A16) $${∥\left(\begin{array}{c} w_{t+1}-w_{t+1}^{\prime }\\ v_{t+1}-v_{t+1}^{\prime } \end{array}\right)∥}_{2}^{2}\leq {∥\left(\begin{array}{c} w_{t}-w_{t}^{\prime }\\ v_{t}-v_{t}^{\prime } \end{array}\right)∥}_{2}^{2}+8L^{2}\eta_{t}^{2}.$$

When $i_{t}=n\;\mathrm{or}\;j_{t}=n,i_{t}\neq j_{t}$, we have

(A17) $$\begin{array}{c} ∥\;\left(\;\begin{array}{c} w_{t+1}-w_{t+1}^{\prime }\\ v_{t+1}-v_{t+1}^{\prime } \end{array}\;\right)\;{∥}_{2}^{2}\;\leq \;∥\;\left(\;\begin{array}{c} w_{t}-\eta_{t}\nabla_{w}\ell w_{t},v_{t};z_{i_{t}},z_{j_{t}})-w_{t}^{\prime }+\eta_{t}\nabla_{w}\ell w_{t}^{\prime },v_{t}^{\prime };z_{i_{t}}^{\prime },z_{j_{t}}^{\prime })\\ v_{t}+\eta_{t}\nabla_{v}\ell \left(w_{t},v_{t};z_{i_{t}},z_{j_{t}}\right)-v_{t}^{\prime }-\eta_{t}\nabla_{v}\ell \left(w_{t}^{\prime },v_{t}^{\prime };z_{i_{t}}^{\prime },z_{j_{t}}^{\prime }\right) \end{array}\;\right)\;{∥}_{2}^{2}\\ \;\leq \;\left(1+p\right)\;∥\;\left(\;\begin{array}{c} w_{t}-w_{t}^{\prime }\\ v_{t}-v_{t}^{\prime } \end{array}\;\right)\;{∥}_{2}^{2}+\left(1+\frac{1}{p}\right)\eta_{t}^{2}∥\left(\begin{array}{c} \nabla_{w}\ell \left(w_{t},v_{t};z_{i_{t}},z_{j_{t}}\right)-\nabla_{w}\ell \left(w_{t}^{\prime },v_{t}^{\prime };z_{i_{t}}^{\prime },z_{j_{t}}^{\prime }\right)\\ \nabla_{v}\ell \left(w_{t},v_{t};z_{i_{t}},z_{j_{t}}\right)-\nabla_{v}\ell \left(w_{t}^{\prime },v_{t}^{\prime };z_{i_{t}}^{\prime },z_{j_{t}}^{\prime }\right) \end{array}\right){∥}_{2}^{2}\\ \leq \left(1+p\right)∥\left(\begin{array}{c} w_{t}-w_{t}^{\prime }\\ v_{t}-v_{t}^{\prime } \end{array}\right){∥}_{2}^{2}+8\left(1+1/p\right)\eta_{t}^{2}L^{2}, \end{array}$$

where in the second inequality, we use that, for any p>0, we have ${\left(c+d\right)}^{2}\leq \left(1+p\right)c^{2}+\left(1+1/p\right)d^{2}$. Combining Equations (A16) and (A17), this gives

$$\begin{array}{c} {∥\left(\begin{array}{c} w_{t+1}-w_{t+1}^{\prime }\\ v_{t+1}-v_{t+1}^{\prime } \end{array}\right)∥}_{2}^{2}\leq \left({∥\left(\begin{array}{c} w_{t}-w_{t}^{\prime }\\ v_{t}-v_{t}^{\prime } \end{array}\right)∥}_{2}^{2}+8L^{2}\eta_{t}^{2}\right)I\left[i_{t}\neq n\;\mathrm{and}\;j_{t}\neq n\right]+\\ \left(\left(1+p\right){∥\left(\begin{array}{c} w_{t}-w_{t}^{\prime }\\ v_{t}-v_{t}^{\prime } \end{array}\right)∥}_{2}^{2}+8\left(1+1/p\right)\eta_{t}^{2}L^{2}\right)I\left[i_{t}=n\;\;\mathrm{or}\;\;j_{t}=n\right]\\ \leq \left(1+pI[i_{t}=n\;\;\mathrm{or}\;\;j_{t}=n]\right){∥\left(\begin{array}{c} w_{t}-w_{t}^{\prime }\\ v_{t}-v_{t}^{\prime } \end{array}\right)∥}_{2}^{2}+8L^{2}\eta_{t}^{2}\left(1+I[i_{t}=n\;\;\mathrm{or}\;\;j_{t}=n]/p\right). \end{array}$$

We apply the above inequality recursively and follow the analysis of Equation (C.4) in Lei et al. [37]:

$$\begin{array}{cc} \; & {∥\left(\begin{array}{c} w_{t+1}-w_{t+1}^{\prime }\\ v_{t+1}-v_{t+1}^{\prime } \end{array}\right)∥}_{2}^{2}\\ \leq & 8L^{2}\eta^{2}\sum_{k=1}^{t}\left(1+I[i_{k}=n\;\;\mathrm{or}\;\;j_{k}=n]/p\right)\prod_{r=k+1}^{t}\left(1+pI[i_{r}=n\;\;\mathrm{or}\;\;j_{r}=n]\right)\\ = & 8L^{2}\eta^{2}\sum_{k=1}^{t}\left(1+I[i_{k}=n\;\;\mathrm{or}\;\;j_{k}=n]/p\right)\prod_{r=k+1}^{t}{\left(1+p\right)}^{I[i_{r}=n\;\;\mathrm{or}\;\;j_{r}=n]}\\ \leq & 8L^{2}\eta^{2}{\left(1+p\right)}^{\sum_{k=1}^{t}I\left[i_{k}=n\;\mathrm{or}\;j_{k}=n\right]}\left(t+\sum_{k=1}^{t}I\left[i_{k}=n\;\mathrm{or}\;j_{k}=n\right]/p\right), \end{array}$$

where we assume fixed step sizes. We set $p=1/\sum_{k=1}^{t}I\left[i_{k}=n\;\mathrm{or}\;j_{k}=n\right]$ and use the inequality ${\left(1+1/x\right)}^{x}\leq e$ to derive

$${∥\left(\begin{array}{c} w_{t+1}-w_{t+1}^{\prime }\\ v_{t+1}-v_{t+1}^{\prime } \end{array}\right)∥}_{2}^{2}\leq 8eL^{2}\eta^{2}\left(t+{\left(\sum_{k=1}^{t}I\left[i_{k}=n\;\mathrm{or}\;j_{k}=n\right]\right)}^{2}\right).$$

It then follows that

$${∥\left(\begin{array}{c} w_{t+1}-w_{t+1}^{\prime }\\ v_{t+1}-v_{t+1}^{\prime } \end{array}\right)∥}_{2}\leq \sqrt{8e}L\eta \left(\sqrt{t}+\sum_{k=1}^{t}I\left[i_{k}=n\;\mathrm{or}\;j_{k}=n\right]\right).$$

By *L*-Lipschitzness, we have

$$\begin{array}{c} |\ell \left(A_{w}\left(S;\phi \right),A_{v}\left(S;\phi \right),z,\tilde{z}\right)-\ell \left(A_{w}\left(S^{\prime };\phi \right),A_{v}\left(S^{\prime };\phi \right),z,\tilde{z}\right)|\\ \leq 2\sqrt{2e}L^{2}\eta \left(\sqrt{t}+\max_{k\in [n]}\sum_{r=1}^{t}I\left[i_{r}=k\;\mathrm{or}\;j_{r}=k\right]\right). \end{array}$$

Therefore, we know that Pairwise SGDA is $\beta_{\phi }$-uniformly stable with

(A18) $$\beta_{\phi }=2\sqrt{2e}L^{2}\eta \left(\sqrt{t}+\max_{k\in [n]}\sum_{r=1}^{t}I\left[i_{r}=k\;\mathrm{or}\;j_{r}=k\right]\right).$$

For simplicity, let $\beta_{\phi ,k}=2\sqrt{2e}L^{2}\eta \left(\sqrt{t}+\sum_{r=1}^{t}I\left[i_{r}=n\;\mathrm{or}\;j_{r}=n\right]\right)$. Applying Lemma A4 to Equation (A18), with probability of at least 1−δ/n, we have

$$\begin{array}{c} \beta_{\phi ,k}\leq 2\sqrt{2e}L^{2}\eta \left(\sqrt{t}+2t/n+\log\left(n/\delta \right)+2\sqrt{t/n\log(n/\delta )}\right). \end{array}$$

With probability of at least 1−δ, the following holds for all k∈[n]:

$$\beta_{\phi ,k}\leq 2\sqrt{2e}L^{2}\eta \left(\sqrt{t}+2t/n+\log\left(n/\delta \right)+2\sqrt{t/n\log(n/\delta )}\right).$$

This suggests the following inequality with probability of at least 1−δ:

$$\beta_{\phi }\leq 2\sqrt{2e}L^{2}\eta \left(\sqrt{t}+2t/n+2\log\left(1/\delta \right)+2\sqrt{2t/n\log(1/\delta )}\right).$$

This suggests that Pairwise SGDA with uniform sampling distribution and the hyperparameter ϕ satisfies sub-exponential stability (Definition 2) with

$$c_{1}=2\sqrt{e}L^{2}\eta \left(\sqrt{t}+2t/n\right),\;c_{2}=4\sqrt{2e}L^{2}\eta \left(1+\sqrt{2t/n}\right).$$

The proof is completed. □

**Proof** **of Lemma 3, (2).** Without loss of generality, we first assume *S* and $S^{\prime }$ differ by the last example. Based on Lemma A5 (2), if $i_{t}\neq n\;\mathrm{and}\;j_{t}\neq n$, we have

$$\begin{array}{c} ∥\left(\begin{array}{c} w_{t+1}-w_{t+1}^{\prime }\\ v_{t+1}-v_{t+1}^{\prime } \end{array}\right){∥}_{2}^{2}=∥\left(\begin{array}{c} w_{t}-\eta_{t}\nabla_{w}\ell \left(w_{t},v_{t};z_{i_{t}},z_{j_{t}}\right)\\ v_{t}+\eta_{t}\nabla_{v}\ell \left(w_{t},v_{t};z_{i_{t}},z_{j_{t}}\right) \end{array}\right)-\\ \left(\begin{array}{c} w_{t}^{\prime }-\eta_{t}\nabla_{w}\ell \left(w_{t}^{\prime },v_{t}^{\prime };z_{i_{t}},z_{j_{t}}\right)\\ v_{t}^{\prime }+\eta_{t}\nabla_{v}\ell \left(w_{t}^{\prime },v_{t}^{\prime };z_{i_{t}},z_{j_{t}}\right) \end{array}\right){∥}_{2}^{2}\leq \left(1+\alpha^{2}\eta_{t}^{2}\right)∥\left(\begin{array}{c} w_{t}-w_{t}^{\prime }\\ v_{t}-v_{t}^{\prime } \end{array}\right){∥}_{2}^{2}. \end{array}$$

When $i_{t}=n\;\mathrm{or}\;j_{t}=n$, we consider Equation (A17). Combining these two cases, we get

(A19) $$\begin{array}{c} ∥\left(\begin{array}{c} w_{t+1}-w_{t+1}^{\prime }\\ v_{t+1}-v_{t+1}^{\prime } \end{array}\right){∥}_{2}^{2}\leq \left(1+\alpha^{2}\eta_{t}^{2}\right)∥\left(\begin{array}{c} w_{t}-w_{t}^{\prime }\\ v_{t}-v_{t}^{\prime } \end{array}\right){∥}_{2}^{2}I\left[i_{t}\neq n\;\mathrm{and}\;j_{t}\neq n\right]+\\ \left(\left(1+p\right)∥\left(\begin{array}{c} w_{t}-w_{t}^{\prime }\\ v_{t}-v_{t}^{\prime } \end{array}\right){∥}_{2}^{2}+8\left(1+\frac{1}{p}\right)\eta_{t}^{2}L^{2}\right)I\left[i_{t}=n\;\;\mathrm{or}\;\;j_{t}=n\right]\\ \leq \left(1+\alpha^{2}\eta_{t}^{2}pI\left[i_{t}=n\;\;\mathrm{or}\;\;j_{t}=n\right]\right)∥\left(\begin{array}{c} w_{t}-w_{t}^{\prime }\\ v_{t}-v_{t}^{\prime } \end{array}\right){∥}_{2}^{2}+8\left(1+\frac{1}{p}\right)\eta_{t}^{2}L^{2}I\left[i_{t}=n\;\;\mathrm{or}\;\;j_{t}=n\right]. \end{array}$$

We apply the above Equation (A19) recursively, following the proof of Theorem 2(d) in Lei et al. [37],

$$\begin{array}{cc} \; & ∥\left(\begin{array}{c} w_{t+1}-w_{t+1}^{\prime }\\ v_{t+1}-v_{t+1}^{\prime } \end{array}\right){∥}_{2}^{2}\\ \leq & 8\left(1+1/p\right)L^{2}\sum_{k=1}^{t}\eta_{k}^{2}I\left[i_{k}=n\;\;\mathrm{or}\;\;j_{k}=n\right]\prod_{r=k+1}^{t}\left(1+\alpha^{2}\eta_{r}^{2}+pI\left[i_{r}=n\;\;\mathrm{or}\;\;j_{r}=n\right]\right)\\ \leq & 8\left(1+\frac{1}{p}\right)L^{2}\eta^{2}\sum_{k=1}^{t}I\left[i_{k}=n\;\;\mathrm{or}\;\;j_{k}=n\right]\prod_{r=k+1}^{t}\left(1+\alpha^{2}\eta_{r}^{2}\right)\prod_{r=k+1}^{t}\left(1+pI\left[i_{r}=n\;\;\mathrm{or}\;\;j_{r}=n\right]\right)\\ = & 8\left(1+1/p\right)L^{2}\eta^{2}\sum_{k=1}^{t}I\left[i_{k}=n\;\;\mathrm{or}\;\;j_{k}=n\right]\prod_{r=k+1}^{t}\left(1+\alpha^{2}\eta_{r}^{2}\right)\prod_{r=k+1}^{t}{\left(1+p\right)}^{I[i_{r}=n\;\;\mathrm{or}\;\;j_{r}=n]}\\ \leq & 8\left(1+1/p\right)L^{2}\eta^{2}\prod_{k=1}^{t}\left(1+\alpha^{2}\eta_{k}^{2}\right)\prod_{k=1}^{t}{\left(1+p\right)}^{I\left[i_{k}=n\;\mathrm{or}\;j_{k}=n\right]}\sum_{k=1}^{t}I\left[i_{k}=n\;\mathrm{or}\;j_{k}=n\right]\\ \leq & 8\left(1+1/p\right)L^{2}\eta^{2}\exp\left(\alpha^{2}\sum_{k=1}^{t}\eta_{k}^{2}\right){\left(1+p\right)}^{\sum_{k=1}^{t}I\left[i_{k}=n\;\mathrm{or}\;j_{k}=n\right]}\sum_{k=1}^{t}I\left[i_{k}=n\;\mathrm{or}\;j_{k}=n\right], \end{array}$$

where we assume fixed step sizes and use $1+x\leq e^{x}$ in the last inequality. We set $p=1/\sum_{k=1}^{t}I\left[i_{k}=n\;\;\mathrm{or}\;\;j_{k}=n\right]$ and use the inequality ${\left(1+1/x\right)}^{x}\leq e$ to derive

$${∥\left(\begin{array}{c} w_{t+1}-w_{t+1}^{\prime }\\ v_{t+1}-v_{t+1}^{\prime } \end{array}\right)∥}_{2}^{2}\leq 8e{\left(1+\sum_{k=1}^{t}I\left[i_{k}=n\;\mathrm{or}\;j_{k}=n\right]\right)}^{2}L^{2}\eta^{2}\exp\left(\alpha^{2}\sum_{k=1}^{t}\eta_{k}^{2}\right).$$

Based on *L*-Lipschitzness and the above inequality, for any two neighboring datasets $S,S^{\prime }\in Z^{n},\forall z,\tilde{z}\in Z,$ we have

$$\begin{array}{c} |\ell \left(A_{w}\left(S;\phi \right),A_{v}\left(S;\phi \right),z,\tilde{z}\right)-\ell \left(A_{w}\left(S^{\prime };\phi \right),A_{v}\left(S^{\prime };\phi \right),z,\tilde{z}\right)|\\ \leq 4\sqrt{e}L^{2}\eta \exp\left(\frac{1}{2}\alpha^{2}t\eta^{2}\right)\max_{k\in [n]}\left(1+\sum_{r=1}^{t}I\left[i_{r}=k\;\mathrm{or}\;j_{r}=k\right]\right). \end{array}$$

Therefore, we know that Pairwise SGDA is $\beta_{\phi }$-uniformly stable with

$$\begin{array}{c} \beta_{\phi }=4\sqrt{e}L^{2}\eta \exp\left(\frac{1}{2}\alpha^{2}t\eta^{2}\right)\max_{k\in [n]}\left(1+\sum_{r=1}^{t}I\left[i_{r}=k\;\mathrm{or}\;j_{r}=k\right]\right). \end{array}$$

For simplicity, let $\beta_{\phi ,k}=4\sqrt{e}L^{2}\eta \exp\left(\frac{1}{2}\alpha^{2}t\eta^{2}\right)\left(1+\sum_{r=1}^{t}I\left[i_{r}=k\;\mathrm{or}\;j_{r}=k\right]\right)$ for any k∈[n]. Taking the expectation over both sides of the above inequality, we derive

(A20) $$c_{1}=4\sqrt{e}L^{2}\eta \exp\left(\frac{1}{2}\alpha^{2}t\eta^{2}\right)\left(1+2t/n\right),$$

where $E[I\left[i_{r}=k\;\mathrm{or}\;j_{r}=k\right]]\leq 2/n.$ Applying $Z_{r}=I\left[i_{r}=k\;\mathrm{or}\;j_{r}=k\right]$ in Lemma A4, we get the following inequality with probability of at least 1−δ/n:

(A21) $$\begin{array}{cc} \beta_{\phi ,k}\; & \leq 4\sqrt{e}L^{2}\eta \exp\left(\frac{1}{2}\alpha^{2}t\eta^{2}\right)\left(1+2t/n+\log\left(n/\delta \right)+2\sqrt{t/n\log(n/\delta )}\right). \end{array}$$

By the union bound in probability, with probability of at least 1−δ, Equation (A21) holds for all k∈[n]. Therefore, with probability of at least 1−δ,

$$\begin{array}{cc} \beta_{\phi } & \leq 4\sqrt{e}L^{2}\eta \exp\left(\frac{1}{2}\alpha^{2}t\eta^{2}\right)\left(1+2t/n+\log\left(n/\delta \right)+2\sqrt{t/n\log(n/\delta )}\right)\\ \; & \leq 4\sqrt{e}L^{2}\eta \exp\left(\frac{1}{2}\alpha^{2}t\eta^{2}\right)\left(1+2t/n+2\log\left(1/\delta \right)+2\sqrt{2t/n\log(1/\delta )}\right)\\ \; & \leq 4\sqrt{e}L^{2}\eta \exp\left(\frac{1}{2}\alpha^{2}t\eta^{2}\right)\left(1+2t/n\right)+8\sqrt{e}L^{2}\eta \exp\left(\frac{1}{2}\alpha^{2}t\eta^{2}\right)\left(1+\sqrt{2t/n}\right)\log\left(1/\delta \right)\\ \; & \leq c_{1}+8\sqrt{e}L^{2}\eta \exp\left(\frac{1}{2}\alpha^{2}t\eta^{2}\right)\left(1+\sqrt{2t/n}\right)\log\left(1/\delta \right), \end{array}$$

where we have used δ∈(0,1/n) in the second inequality and Equation (A20) in the last inequality. Therefore, sub-exponential stability (Definition 2) holds with $c_{1}=4\sqrt{e}L^{2}\eta \exp\left(\frac{1}{2}\alpha^{2}t\eta^{2}\right)\left(1+2t/n\right)$ and $c_{2}=8\sqrt{e}L^{2}\eta \exp\left(\frac{1}{2}\alpha^{2}t\eta^{2}\right)\left(1+\sqrt{2t/n}\right)$. The proof is completed. □

Based on the above lemma, we are ready to develop generalization bounds in Theorem 2 for Pairwise SGDA with smooth and non-smooth loss functions.

**Proof** **of Theorem 2.** With $A\left(S;\phi \right)=\left(A_{w}\left(S;\phi \right),A_{v}\left(S;\phi \right)\right)$, based on Lemma 3, (1) and (2), Pairwise SGDA with both convex-concave non-smooth as well as convex-concave smooth loss functions satisfies sub-exponential stability (Definition 2). Applying the upper bounds on $\beta_{\phi }$ to Lemma 1, we obtain the result. □
